# Pharmacological Targets for Periodontitis: A Systematic Review of Drug‐Target Mendelian Randomisation Studies

**DOI:** 10.1111/jre.70168

**Published:** 2026-09-02

**Authors:** Luigi Nibali, Jing Kang, Simon Haworth, Alessandro Polizzi, Gaetano Isola

**Affiliations:** ^1^ Periodontology Unit, Centre for Host Microbiome Interactions, Faculty of Dentistry, Oral & Craniofacial Sciences King's College London London UK; ^2^ Centre for Oral, Clinical and Translational Sciences, Faculty of Dentistry, Oral & Craniofacial Sciences King's College London London UK; ^3^ Bristol Dental School University of Bristol Bristol UK; ^4^ Department of General Surgery and Surgical‐Medical Specialties, School of Dentistry University of Catania Catania Italy; ^5^ International Research Center on Periodontal and Systemic Health ‘PerioHealth’ University of Catania Catania Italy

**Keywords:** cytokine, drug, inflammation, mendelian, periodontitis

## Abstract

**Aim:**

Drug‐target Mendelian randomisation (MR) studies investigate whether genetically proxied modulation of druggable proteins affects the risk of periodontitis, thereby identifying potential pharmacological targets.

**Methods:**

A systematic review was conducted following PRISMA 2020 guidelines. Three electronic databases (MEDLINE/Pubmed, Scopus, Web of Science) were searched. Eligible studies applied MR to evaluate causal relationships between druggable targets and periodontitis. Data on study design, population, exposure, and outcomes were extracted; risk of bias was assessed qualitatively. Supplementary searches of Google Scholar, reference lists, and medRxiv were also undertaken.

**Results:**

Fifteen MR studies published between 2023 and 2026 were included. Underlying data on periodontal outcomes were derived from a limited number of studies. Identified targets spanned inflammatory cytokines (e.g., IL6R), complement components (C3 and C5), immune regulators (CXCL10 and CXCR4), calcium‐binding proteins (S100A8/A9/A12), and proteins involved in angiogenesis, apoptosis, cell‐cycle regulation, and extracellular‐matrix remodelling, including VEGFA, CASP3, CCND1, and MMP25. Several studies integrated multi‐omics, colocalization, and single‐cell approaches. However, the therapeutic relevance of these targets remains to be established through independent replication and experimental or clinical validation.

**Conclusion:**

Drug‐target MR provides a platform for investigating host molecular traits linked to periodontal disease and highlights multiple targets for possible drug repurposing in the future. Methodological and data constraints currently limit the validity of these findings and their potential clinical applications.

## Graphical abstract

This systematic review evaluated drug‐target Mendelian randomisation studies on periodontitis. Twelve studies identified host‐response‐related targets mainly involved in inflammatory, complement, and immune pathways. Although some targets were linked to existing drugs, the evidence remains preliminary and heterogeneous, requiring replication and further validation before clinical application.

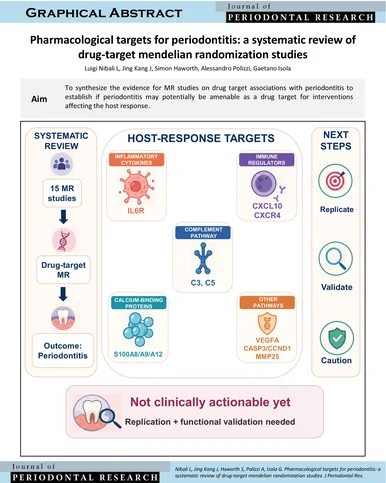

## 1 Introduction

Periodontitis is a complex, multifactorial disease of bacterial origin which is followed by a host response that has a fundamental role in disease onset and progression [1]. Over the last decades, similarities and associations have emerged between periodontitis and chronic non‐communicable diseases (NCDs) characterised by aberrant host response, such as rheumatoid arthritis and inflammatory bowel diseases [2]. It is now clear that periodontitis is one of a number of NCDs that share many common denominators and pathological pathways [3]. As such, although periodontitis is normally treated very effectively by mechanical strategies that limit the subgingival biofilm growth [4], the use of medications as adjuncts has also been considered. These drugs mainly consist of chemical adjuncts, primarily antiseptics and antibiotics [5, 6], although medications affecting inflammation and host response have also been used [7, 8].

Although not a new concept, in recent years, there has been increasing recognition that periodontitis should be understood not only as a local infection‐driven condition but also as a systemic inflammatory disease with a strong genetic component [9]. Genome‐wide association studies (GWAS) and several other investigations have highlighted several loci linked to immune regulation and inflammatory pathways that predispose to periodontitis [10, 11, 12]. However, no periodontitis‐specific loci have been identified, which may reflect measurement limitations or the polygenic and systemic nature of the disease. This evidence supports the concept that host susceptibility is a key determinant of disease initiation and progression, and it provides a rationale for exploring molecular pathways that may be therapeutically modulated [13]. At the same time, pharmacogenomic and drug repurposing strategies have gained traction in periodontology [14]. The idea that drugs originally developed for cardiovascular, metabolic, or autoimmune diseases could be repurposed for prevention and potentially management of periodontitis has become particularly appealing, since many of the underlying biological pathways are shared [15].

Despite the growing interest in drug targets for periodontitis, there are currently few examples of successful drug repurposing [16] and there is a need to identify additional targets in an evidence‐based and systematic manner. Within this framework, Mendelian randomisation (MR) studies have emerged as a powerful tool to assess causality between druggable targets and periodontitis [17, 18]. Drug‐target MR uses single‐nucleotide polymorphisms (SNPs) which influence a druggable protein, estimating whether changing the protein may alter disease expression [19]. By leveraging genetic variants as natural experiments, MR is more robust to the problems of confounding and reverse causality which are problematic in classical observational studies [20]. Specifically, drug‐target MR uses cis‐acting or target‐proximal genetic variants to evaluate molecular traits within an explicit pharmacological‐target framework, encompassing both focused validation of established targets and systematic discovery of candidate therapeutic targets [21]. This approach has potential to provide valuable information for prioritising candidate targets and predicting both beneficial and adverse effects of pharmacological intervention.

The aim of this systematic review is to synthesise the evidence for MR studies on drug target associations with periodontitis to ultimately try to establish if periodontitis may potentially be amenable as a drug target for interventions affecting the host response.

## 2 Methods

### 2.1 Protocol Development and Focused Question

The systematic review was registered in PROSPERO (registration number: CRD420251149757). No protocol amendments have been made after registration. It is reported using the Preferred Reporting Items for Systematic Review and Meta‐Analyses (PRISMA 2020) guidelines [22]. The PRISMA statement is available in Appendix S1

The primary objective of this systematic review was to appraise the existing literature on MR studies on drug target associations with periodontitis by answering the following focused question: “Could periodontitis be amenable as a drug target for interventions affecting the host response?”

The research question was formulated based on the following PECOS criteria:

Pecos:

- Population: Adult populations with data on periodontal status
- Exposure: Genetically proxied drug targets
- Outcome: Presence/severity of periodontitis
- Comparison: Periodontal health (or different levels of periodontal severity)
- Study design: Mendelian randomisation studies

### 2.2 Eligibility Criteria

The eligibility criteria were the following:

- Studies adopted an MR analytical framework using single‐nucleotide polymorphisms to instrument the molecular exposure;
- The genetic instruments were restricted to cis‐acting or target‐proximal variants for the corresponding gene or protein;
- Pharmacological‐target validation or discovery was an explicit component of the study design;
- The outcome variable related to periodontal status

Eligible designs comprised: (i) focused cis‐MR analyses of established or prespecified individual drug targets; (ii) systematic cis‐QTL analyses restricted a priori to curated sets of druggable genes or proteins; and (iii) proteome‐wide cis‐pQTL analyses explicitly designed to identify potential therapeutic targets and incorporating a downstream assessment of druggability. Studies using trans or unrestricted genome‐wide instruments for circulating molecular traits, and broader disease‐gene discovery analyses without an explicit pharmacological‐target framework, were excluded.

### 2.3 Search Methods

The search strategy was designed to cover all potentially suitable publications. The electronic databases searched included MEDLINE (Ovid), Scopus and Web of Science (WoS), up to April 2026, with no limitations on publication year. The search combined terms related to periodontitis, Mendelian randomisation methodology, and drug‐target prioritisation, including SMR, colocalization, cis‐MR, pQTL, eQTL, proteome‐wide, drug repurposing, and target prioritisation. Full database‐specific search strings and the number of retrieved records are reported in the Table S1.

In addition, relevant narrative and systematic reviews were searched and assessed to identify suitable papers. The results from all databases were combined, and duplicates were eliminated. To maximise the inclusion of pertinent studies, the bibliographies of papers that met the eligibility criteria were also reviewed, and a manual search on Google Scholar was performed to find additional relevant references, which underwent the same screening and selection process. A targeted search of medRxiv was additionally conducted in July 2026 to identify potentially eligible preprints using combinations of the terms “periodontitis”, “Mendelian randomisation” or “Mendelian randomisation”, “drug target”, “druggable”, “cis‐MR”, “eQTL”, and “pQTL”. Retrieved records were screened against the same eligibility criteria.

### 2.4 Study Selection

Two independent reviewers (authors A.P. and G.I.) carried out the first stage of study selection by screening all titles and abstracts against the inclusion criteria. The resulting database with studies included by at least one reviewer was subjected to second‐stage screening, carried out by authors A.P. and G.I., during which full‐text versions of papers identified as possibly relevant were retrieved and screened against the inclusion criteria; studies not meeting the inclusion criteria were excluded (Table S2). In the presence of disagreement between reviewers, study eligibility was attempted by discussion or with the help of an arbitrator (author L.N.). The agreement between reviewers was calculated using kappa statistics for the first‐ and second‐stage screening. For this review, drug‐target Mendelian randomisation was operationally defined as an MR analysis in which cis‐acting or target‐proximal genetic variants were used to proxy modulation of a molecular target and pharmacological‐target validation or discovery was an explicit component of the analytical design. This definition included both focused analyses of individual targets and systematic cis‐QTL target‐discovery studies, but excluded analyses relying on trans or unrestricted genome‐wide instruments and broader gene‐expression MR studies lacking an explicit drug‐target framework.

### 2.5 Data Collection

A data extraction form was developed to extract study characteristics. The following data were extracted:

- Study characteristics: Author, year, source of publication, funding and setting (country, hospital, university, community, other).
- Population: The number of participants, demographics (age, gender, ancestry, smoking, socioeconomic factors, body mass index/obesity), periodontal conditions (health/periodontitis or different severities of periodontitis)
- Exposure: Genetically proxied drug targets on the same PECOS populations
- Instrument‐selection characteristics: definition and width of the cis‐region, association threshold used for instrument selection, number of retained instruments, linkage‐disequilibrium clumping or correlation‐handling criterion, and reported measures of instrument strength.
- Outcomes/comparison: definition and diagnosis of periodontitis and/or health

Data on study characteristics, populations, and interventions were transferred into evidence tables to summarise the included studies.

### 2.6 Risk of Bias Assessment

Two reviewers (authors A.P. and G.I.) independently performed a qualitative credibility assessment of the included studies, informed by the principled framework for Mendelian randomisation in oral health research proposed by Bashir et al. [23]. At the study level, MR applicability was defined as the conceptual suitability of using genetically regulated expression or circulating levels of a druggable gene or protein as the exposure in relation to periodontitis. All included studies fulfilled this broad applicability criterion because they used cis‐acting or target‐proximal variants to proxy druggable molecular traits. This judgement was distinct from target‐level gene–environment equivalence, which assessed whether the lifelong genetic perturbation could reasonably approximate the direction, mechanism, tissue context, magnitude, and time course of a pharmacological intervention. Accordingly, an analysis could be considered suitable for MR while its pharmacological equivalence remained questionable or not assessable.

Each study was then evaluated using a five‐item credibility checklist covering: (Q1) instrument relevance, (Q2) biological plausibility of the exchangeability assumption, (Q3) biological plausibility of the exclusion restriction assumption, (Q4) analytical robustness, and (Q5) reporting transparency and interpretability of the periodontitis outcome. Instrument relevance (Q1) was assessed on a study‐specific basis, considering the biological definition and width of the cis‐region, the association threshold used to select instruments, the number of retained variants, reported F‐statistics or variance explained, and the biological or statistical justification for any relaxed selection threshold. No single association threshold was considered mandatory for cis‐MR. Linkage‐disequilibrium handling was considered primarily under analytical robustness (Q4), including the reported clumping threshold and whether correlation between retained variants was appropriately modelled or otherwise accounted for.

The exchangeability and exclusion restriction assumptions were not treated as directly testable. Instead, they were judged qualitatively based on the biological specificity of the selected cis‐acting instruments, the likelihood of alternative pathways linking the variants to the outcome, and whether the original studies reported supporting analyses such as colocalization, phenome‐wide scans, heterogeneity tests, or MR‐Egger intercepts.

Analytical robustness also considered aspects such as LD handling/clumping, harmonisation, replication using secondary datasets where applicable, and the extent of sensitivity analyses. Reporting quality was considered only in terms of transparency and interpretability, including how clearly periodontitis was defined and ascertained, and not as a direct measure of causal validity.

Study‐specific cis‐windows, instrument‐selection thresholds, LD‐handling criteria, and instrument‐strength measures are reported in Table S3 and informed the Q1 and Q4 judgements in Table 3.

Each domain was rated qualitatively as “low”, “some concerns”, or “high” as appropriate. The overall study‐level judgement was derived from the combined pattern across the five items: Low when all domains were Low or only 1 was some concerns; Some concerns when 2–3 domains were some concerns or 1 domain was high; and High when ≥ 4 domains were some concerns or ≥ 2 domains were high. MR applicability was recorded separately and was not included in the five‐item credibility assessment used to derive the overall study‐level judgement.

### 2.7 Data Synthesis

Meta‐analysis was considered to assess the Weighted Mean Difference (WMD) with 95% Confidence Interval (CI) in the presence of a sufficient number of similar studies (at least 3) with non‐overlapping participants.

Formal statistical assessment of publication bias was not performed because no quantitative synthesis was conducted and no target‐outcome association was evaluated by a sufficient number of comparable studies to support funnel‐plot or small‐study‐effect analyses.

A qualitative biological function commonality analysis of the identified drug targets was performed. For each target gene or protein reported in the included MR studies, biological function descriptions and keyword annotations were extracted from UniProtKB (https://www.uniprot.org/) using the Gene Ontology classification system [39]. These annotations were used to summarise shared functional themes across targets. As a qualitative sensitivity analysis, functional commonality was assessed both when including all studies and when restricting the synthesis to studies judged to be at overall low risk of bias.

### 2.8 Certainty of Evidence

We reviewed the overall results for each outcome and assessed the level of certainty in the evidence as high, moderate, low, or very low, using the GRADEpro Guideline Development Tool (McMaster University and Evidence Prime, 2024; gradepro.org) by two independent researchers (JK and LN).

## 3 Results

Figure 1 reports the systematic review flowchart. The initial search strategy resulted in a total of 408 records, and after de‐duplication, 234 records were retained. After the second round of screening, 24 studies were assessed in full‐text for eligibility. Kappa score for reviewers' agreement was 0.90 at first and 1.00 at the second screening. The targeted medRxiv search identified one additional unique eligible preprint after deduplication and eligibility assessment. Therefore, 15 studies were included in the final synthesis.

**FIGURE 1:**
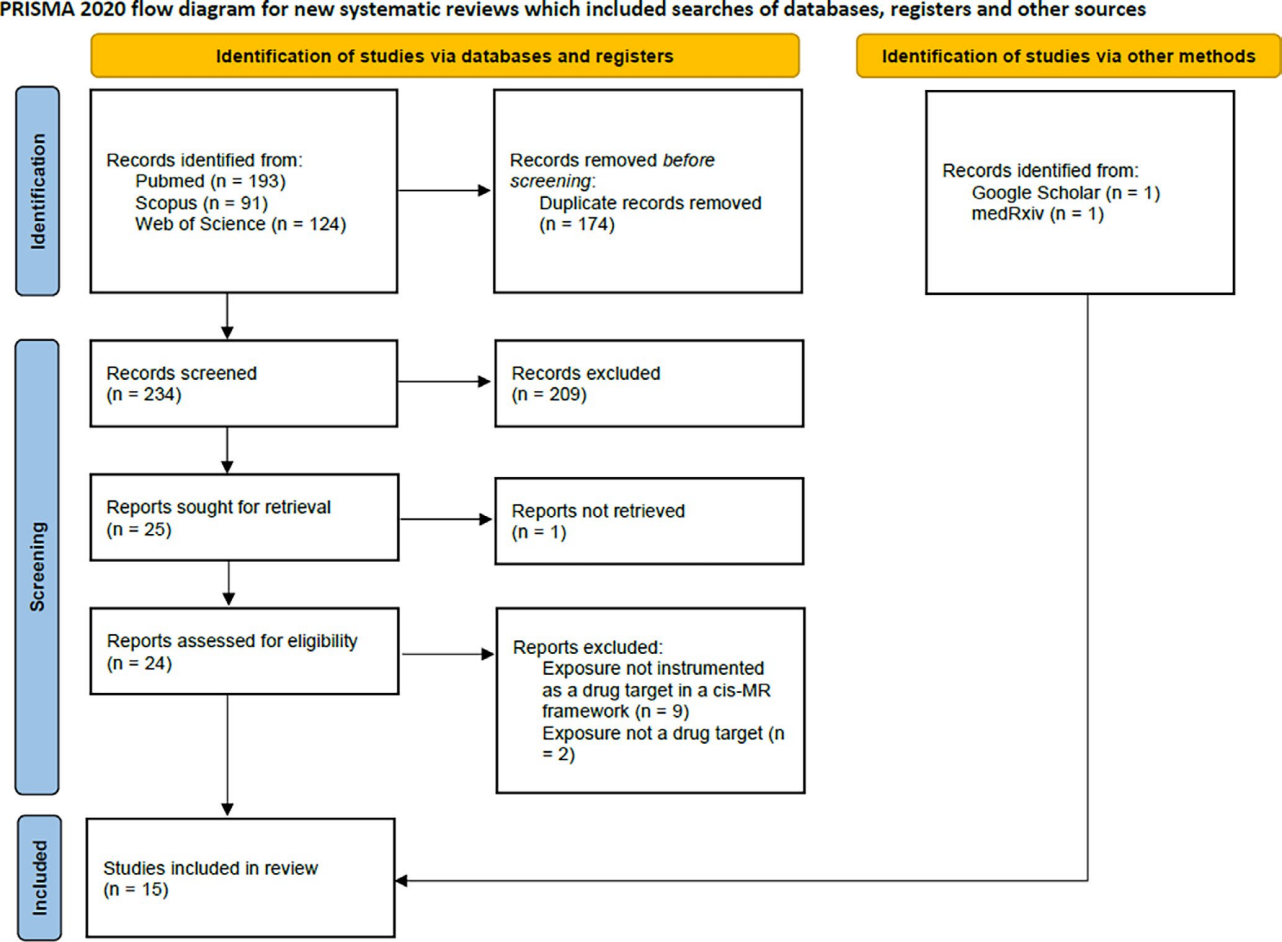
PRISMA flow chart of the study.

Table 1 presents a summary of data from the included papers. The publication date ranged from 2023 to 2026. Among the 15 studies included, design and analysis of eight studies were carried out in mainland China [29, 30, 31, 32, 33, 34, 35, 38], one in Hong Kong [37], and six were conducted by the same research groups in Greifswald and Münster, Germany [24, 25, 26, 27, 28, 36]. Source data were obtained from large GWAS consortia such as GLIDE, FinnGen, and UKBiobank, the Million Veteran Program, deCODE genetics, and eQTLGen, together with blood cis‐eQTL and proteome‐wide pQTL datasets. Several studies combined these with single‐cell RNA sequencing, colocalization, SMR/HEIDI analyses, molecular docking, or broader multi‐omics frameworks. The included evidence comprised both focused validation studies of individual pharmacological targets and systematic target‐discovery studies. The latter included cis‐QTL analyses restricted to curated druggable‐gene sets and proteome‐wide analyses using target‐proximal cis‐pQTL instruments, whereas studies based on trans or unrestricted genome‐wide instruments were not considered drug‐target MR.

**TABLE 1:** Data extracted from the included studies.

Study	Funding and setting	Sample	Exposure (gene/protein target)	Outcome	Methods	Main findings
Alayash et al., 2023 [24]	Institute of Health Services Research in Dentistry, University of Münster, Münster, Germany. No specific funding reported.	Exposure GWAS: C‐reactive protein GWAS meta‐analysis including 575 531 individuals of European ancestry. Outcome GWAS: GLIDE consortium, 17 353 periodontitis cases and 28 210 controls of European ancestry.	Genetically proxied inhibition of tumour necrosis factor receptor 1 (TNFR1), indexed by variants in the vicinity of the TNFRSF1A gene and associated with CRP levels.	Presence of periodontitis (defined in GLIDE using CDC/AAP or CPI case definitions)	Drug‐target cis‐MR using variants near TNFRSF1A. Primary analysis used Wald ratio with rs1800693; secondary analysis used three correlated variants (rs767455, rs4149570, rs4149577) with fixed‐effects IVW accounting for correlation and PCA‐based IVW.	No evidence that genetically proxied TNFR1 inhibition reduced periodontitis risk. Primary analysis with rs1800693 showed OR 1.57 (95% CI 0.38–6.46); secondary analyses using three variants were similarly null.
Alayash et al., 2024 (a) [25]	Institute of Health Services Research in Dentistry, University of Münster, Münster, Germany. No fundings declared.	C3 indexing GWAS derived from 5368 European descent; Periodontitis GWAS derived from GLIDE consortium (17 353 participants of European ancestry were classified as clinical periodontitis cases and 28 210 as controls)	Complement C3	Presence of periodontitis (defined by CDC/AAP or CPI)	cis‐MR approach with three uncorrelated SNPs and IVW meta‐analysis to combine the Wald ratios	The study found a causal association between genetically proxied inhibition of Complement C3 and a reduced risk of periodontitis.
Alayash et al., 2024 (b) [26]	Institute of Health Services Research in Dentistry, University of Münster, Münster, Germany. No fundings declared.	GLIDE consortium (17 353 periodontitis cases, 28 210 healthy controls, European ancestry)	Complement C5	Presence of periodontitis	MR: drug target IV	Genetic inhibition of C5 is associated with reduced risk of periodontitis, showing a rationale for C5 inhibitors in clinical trials
Alayash et al., 2026 [27]	Institute of Health Services Research in Dentistry, University of Münster, Münster, Germany. Funding: Deutsche Forschungsgemeinschaft (DFG, German Research Foundation), Grant 541 494 246.	Exposure: proteome‐wide pQTL study of 4719 circulating proteins in 35 559 Icelanders (deCODE genetics). Outcome GWAS: discovery analysis in the Million Veteran Program (34 270 periodontitis cases and 396 445 controls, European ancestry); replication analysis in the GLIDE consortium (17 353 clinically diagnosed periodontitis cases and 28 210 controls, European ancestry).	Circulating proteins proxied by cis‐pQTLs within ±1 Mb of the corresponding protein‐coding genes; 2088 proteins evaluated after filtering. Main identified targets: FGF2, AZGP1, and BTC.	Presence of periodontitis. Discovery: VA‐MVP Phecode‐based case definition. Replication: GLIDE consortium with CDC/AAP or CPI case definitions.	Proteome‐wide two‐sample cis‐pQTL MR with discovery and replication analyses, followed by FDR correction, Steiger filtering, HEIDI test, colocalisation analysis, druggability assessment, and protein–protein interaction network analysis.	Among 2088 evaluated proteins, three showed robust evidence of causal association with periodontitis: FGF2 (OR 1.06, 95% CI 1.032–1.082), AZGP1 (OR 1.12, 95% CI 1.058–1.189), and BTC (OR 0.86, 95% CI 0.789–0.942). Replication analysis confirmed 18 proteins overall, with 16 showing high colocalisation. None was identified as a direct approved drug target for periodontitis, although indirect links with anti‐inflammatory therapies were reported.
Baumeister et al., 2023 [28]	Institute of Health Services Research in Dentistry, University of Münster, Münster, Germany. No specific funding reported.	Exposure GWAS: LDL cholesterol GWAS meta‐analysis of 188 578 individuals (GLGC); secondary analysis used LDL GWAS data from 115 082 European ancestry participants from the UK Biobank. Outcome GWAS: GLIDE consortium, 17 353 periodontitis cases and 28 210 controls.	Genetically proxied inhibition of HMGCR, NPC1L1, and PCSK9, using variants within 100 kb of the encoding genes associated with LDL cholesterol levels.	Presence of periodontitis (GLIDE consortium; cases defined by CDC/AAP or CPI criteria).	Drug‐target MR using cis‐acting variants near HMGCR, NPC1L1, and PCSK9. Generalised weighted least squares analysis accounted for linkage disequilibrium, with estimates scaled to a 1 mmol/L reduction in LDL cholesterol. Secondary replication analysis used LDL‐effect estimates from UK Biobank.	Genetically proxied inhibition of NPC1L1 and PCSK9 was associated with lower odds of periodontitis, whereas HMGCR inhibition was not. Primary analysis showed OR 0.53 (95% CI 0.35–0.81) for NPC1L1, OR 0.84 (95% CI 0.74–0.95) for PCSK9, and OR 0.92 (95% CI 0.73–1.16) for HMGCR. Secondary analysis confirmed similar findings.
Cheng et al., 2025 [29] (medRxiv preprint)	Zunyi Medical University and collaborating institutions, China. Supported by Zunyi research grants.	eQTLGen blood cis‐eQTL data (318 614 individuals). FinnGen R11 chronic periodontitis GWAS: 5364 cases and 288 472 controls of European ancestry.	A predefined list of 4479 druggable genes; 2288 genes with eligible cis‐eQTL instruments were analysed.	Chronic periodontitis defined using FinnGen registry codes.	Cis‐eQTLs within ±100 kb; *p* < 5 × 10^−8^; LD r2 < 0.1; F > 10. Two‐sample MR with FDR correction, SMR/HEIDI, Bayesian colocalization, FUSION TWAS, and PheWAS‐MR.	Thirteen genes passed FDR correction. MMP25 received the strongest support across MR, SMR/HEIDI, colocalization, and TWAS. TNFRSF18, CDC25B, STK10, and ACVR2B were supported by MR, SMR/HEIDI, and TWAS but not colocalization.
Hu et al., 2025 [30]	Multi‐institutional study conducted in China. Funding: National Natural Science Foundation of China (Grant 82 071 094).	Blood cis‐eQTLs from eQTLGen. Acute periodontitis outcome from FinnGen R10 (367 cases and 195 395 controls; European ancestry).	DrugBank‐defined targets of glucocorticoids and NSAIDs; periodontitis‐associated targets CASP3 and CCND1.	Acute periodontitis.	Two‐stage MR framework. Target‐gene analysis used blood‐derived cis‐eQTLs within ±100 kb, primarily IVW with heterogeneity, pleiotropy, and leave‐one‐out analyses; two‐step mediation MR assessed gut microbiota.	Higher genetically proxied CASP3 expression (OR 1.25, 95% CI 1.09–1.43) and CCND1 expression (OR 1.55, 95% CI 1.33–1.81) were associated with acute periodontitis (both *p* < 0.001); CCND1 showed a potential microbiota‐mediated pathway.
Huoshen et al., 2025 [31]	The Affiliated Stomatology Hospital, South west Medical University, Luzhou, Sichuan, China. Fundings: College Students' Innovative Entrepreneurial Training Plan Program, Grant/Award Number: S202210632228 and S202210632171.	GLIDE consortium (17 353 cases, 28 210 controls, European ancestry)	4463 druggable genes tested; 14 significant (S100A12, S100A9, S100A8, ADAM12, ADHFE1, BLK, HEBP1, SERPINE2, TEK, LY86, MMEL1, S100B, SPP1, TRIB3)	Presence of periodontitis	Curated druggable‐genome cis‐eQTL target‐discovery analysis: cis‐eQTL instruments for genes selected from an a priori druggable‐gene set, followed by SMR and colocalization analyses	14 drug targets identified; 3 strong evidence from same protein family (S100A12, S100A9, S100A8); 6 moderate (ADAM12, ADHFE1, BLK, HEBP1, SERPINE2, and TEK); 5 lower tier (LY86, MMEL1, S100B, SPP1, and TRIB3). Potential repurposing of existing drugs (e.g., labetalol, colchicine, dexamethasone)
Huoshen et al., 2026 [32]	Southwest Medical University and collaborating Chinese institutions. Internal institutional research funding.	FAERS: 38129121 adverse‐event reports, including 5938 periodontitis reports. Protein instruments: UKB‐PPP and deCODE cis‐pQTL datasets. Periodontitis outcomes: FinnGen and GLIDE GWAS; gingival and peripheral‐blood scRNA‐seq datasets.	DrugBank‐mapped protein targets of five drugs with positive FAERS signals; 34 targets evaluated, with VEGFA prioritised.	Presence of periodontitis in pharmacovigilance records and GWAS datasets.	FAERS disproportionality analyses and adjusted logistic regression; DrugBank mapping, enrichment and PPI analysis; cross‐cohort SMR meta‐analysis; two‐sample cis‐pQTL MR; scRNA‐seq validation.	Five drugs showed positive FAERS signals. VEGFA was nominally associated with periodontitis in the SMR meta‐analysis (OR 1.043, 95% CI 1.000–1.089; *p* = 0.049), with positive two‐sample MR associations in FinnGen and GLIDE and prominent expression in monocytes and gingival cell populations.
Jiang et al., 2026 [33]	Department of Stomatology, Shanghai General Hospital, Shanghai Jiaotong University School of Medicine, Shanghai, China. Funding: National Natural Science Foundation of China (No. 81970938)	Exposure: cis‐eQTL data of druggable genes. Outcome GWAS: discovery cohort from FinnGen periodontitis GWAS; validation cohort from the GLIDE consortium. Human gingival samples were also used for qRT‐PCR and immunohistochemistry validation.	Genome‐wide druggable cis‐eQTLs. Main validated target: CACNB4; additional MR‐associated genes included PSMA4, GAA, FGF2, AURKAIP1, and ADAM12.	Presence of periodontitis	Integrative drug‐target MR and colocalization analyses using cis‐eQTL and periodontitis GWAS data, followed by SMR/HEIDI validation, qRT‐PCR, immunohistochemistry, drug prediction, molecular docking, and in vitro/in vivo pharmacological analyses.	Six genes were associated with periodontitis in MR analysis, of which CACNB4 and PSMA4 were supported by colocalization. CACNB4 showed consistent evidence across cohorts and experimental validation, with lower expression in periodontitis tissues. Drug prediction and docking identified verapamil and safinamide as candidate drugs targeting CACNB4, and both attenuated osteoclast differentiation and alveolar bone loss in experimental models.
Liu et al., 2024 [34]	State Key Laboratory of Oral Diseases & National Center for Stomatology & National Clinical Research Center for Oral Diseases, West China Hospital of Stomatology, Sichuan University, Chengdu, China. Fundings: National Natural Science Foundation of China (Grant 82 301 106 for Y. Cao and Grant 81 970 936 for D. Huang), Natural Science Foundation of Sichuan province (Grant 2024NSFSC1585 for Y. Cao), and Fundamental Research Funds for the Central Universities and Research and Develop Program, West China Hospital of Stomatology, Sichuan University (Grant RD‐02‐202 409 for Y. Cao).	Blood eQTL data on healthy European subjects (*N* = 31 684). Outcome: Periodontitis GWAS data (Discovery: FinnGen R9; Replication: GLIDE consortium).	Druggable genes (e.g., CXCL10, MYO3B, SLAMF1), proxied by gene expression levels	Presence of Periodontitis (based on FinnGen R9 and GLIDE consortium GWAS data).	cis‐eQTLs as IVs. Main analysis: IVW. Included sensitivity analysis and colocalization	Three replicated druggable genes showed a causal link: CXCL10 and MYO3B were protective (reduced risk), while SLAMF1 was detrimental (increased risk).
Lou et al., 2026 [35]	School & Hospital of Stomatology, Wenzhou Medical University, Wenzhou, China. Funding: Natural Science Foundation of Zhejiang Province (LQ20H020002).	Exposure: blood cis‐eQTL data from the eQTLGen consortium based on 31 684 individuals of European ancestry; 3275 druggable genes retained after intersecting druggable‐gene resources with eQTL data. Outcome GWAS: FinnGen 11 periodontitis dataset including 406 876 individuals of European ancestry (118 404 cases and 288 472 controls). Single‐cell RNA‐seq dataset: GSE171213, comprising periodontal tissue samples from 4 healthy controls and 5 untreated periodontitis patients.	Genome‐wide druggable cis‐eQTLs. Main prioritised targets: OAT and DPP8.	Presence of periodontitis.	Integrative drug‐target MR and colocalization analysis using cis‐eQTL and FinnGen periodontitis GWAS data, followed by phenome‐wide MR, enrichment analysis, single‐cell RNA‐seq, metabolic pathway analysis, pseudotime analysis, intercellular communication analysis, transcription‐factor regulatory network analysis, candidate drug prediction, and molecular docking.	MR analysis identified OAT and DPP8 as candidate therapeutic targets for periodontitis, with colocalization support. Single‐cell analysis showed predominant OAT expression in fibroblasts, particularly in inflammatory and matrix‐remodelling subsets. OAT+ fibroblasts displayed enhanced metabolic activity and central roles in intercellular communication. Candidate drug prediction linked OAT to probenecid, L‐proline, and D‐arginine, and DPP8 to sitagliptin; molecular docking supported these interactions.
Nolde et al., 2023 [36]	Institute of Health Services Research in Dentistry, University of Münster, Münster, Germany. No fundings declared.	575 531 European descent participants from the UK Biobank and CHARGE consortium (for exposure GWAS); 17 353 clinical periodontitis cases and 28 210 European healthy controls from the GLIDE consortium (for outcome GWAS)	Downregulation of interleukin 6 (IL‐6) signalling (gene/protein target: IL6R)	Presence of periodontitis (defined by CDC/AAP or CPI)	cis‐MR approach and IVW meta‐analysis to combine the Wald ratios	The study suggests that genetically proxied downregulation of IL‐6 signalling is causally associated with a lower risk of periodontitis.
Zhan et al., 2024 [37]	Faculty of Dentistry, The University of Hong Kong, Hong Kong, China. Fundings: HKU Libraries Open Access Author Fund sponsored by the HKU Libraries.	30 377 individuals with periodontitis and 346 900 healthy controls from the FinnGen consortium; the summarised GWAS data on circulating proteins were provided by a GWAS of 4719 proteins from 35 559 Icelanders.	1722 circulating proteins	Presence of periodontitis (defined by hospital diagnoses with clinical manifestations such as attachment loss, deep periodontal pockets, and alveolar bone loss)	Proteome‐wide cis‐pQTL target‐discovery MR of 1722 circulating proteins, with external validation, bidirectional analyses, single‐cell follow‐up, and downstream assessment of therapeutic tractability	Genetically predicted circulating levels of von Willebrand factor A domain‐containing 1 (VWA1) were inversely associated with periodontitis. In contrast, the level of growth differentiation factor 15 (GDF15) might be associated with an increased risk of periodontitis. The causal relationships between VWA1, GDF15, and periodontitis were successfully replicated during the external validation process.
Zhou et al., 2026 [38]	School & Hospital of Stomatology, Wenzhou Medical University, Wenzhou, China. Fundings: the Natural Science Foundation of Zhejiang Province (LQ20H020002), the General Research Project of Zhejiang Provincial Department of Education (Y201942047), the Wenzhou Science and Technology Program (Y20180060), and the National College Students Innovation and Entrepreneurship Training Program (202310343037).	Exposure (eQTLs): Blood eQTL summary statistics from eQTL‐Gen healthy patients (*N* = 31 684 individuals). Outcome (Periodontitis): GWAS summary statistics from the FinnGen consortium (97 830 cases, 272 252 controls, European ancestry)	Genetically predicted expression levels of four hub genes: CXCR4, ARHGDIB, PLAT, C19orf10.	Presence of periodontitis	SMR followed by two‐sample MR using the Wald ratio method for validation of hub genes. Multi‐omics approach included eQTLs, bulk RNA‐seq, scRNA‐seq, machine learning (LASSO/Random Forest) for gene selection, and Colocalization analysis	Increased genetically predicted expression levels of all four hub genes (CXCR4, PLAT, ARHGDIB, C19orf10) were found to be causally associated with an increased risk of periodontitis.

Abbreviations: AAP, American Academy of Periodontology; CDC, Centers for Disease Control; cis‐eQTLs, Cis‐expression quantitative trait loci; CPI, Community Periodontal Index; FAERS, Food and Drug Administration Adverse Event Reporting System; GLGC, Global Lipids Genetics Consortium; GLIDE, Gene Lifestyle Interactions in Dental End‐points; GWAS, Genome‐wide association study; HEIDI, Heterogeneity in the independent instrument; HLA, Human leukocyte antigen; IL, Interleukin; IV, Instrumental variable; IVW, Inverse variance weighted; MD, Molecular docking; MR, Mendelian randomisation; PheWAS, Phenome‐wide association study; SMR, Summary‐data‐based Mendelian randomisation; TNF, Tumour necrosis factor; TWAS, Transcriptome‐wide association study; VEGFA, Vascular endothelial growth factor A; WGCNA, Weighted gene co‐expression network analysis.

Table 2 summarises reported gene/protein‐level drug targets, along with the corresponding pharmacological agents. Several targets correspond to drugs already approved for systemic inflammatory or metabolic conditions potentially linked with periodontitis. For instance, IL‐6R targeting agents such as tocilizumab, sarilumab, and satralizumab are widely used in rheumatoid arthritis and other autoimmune diseases, and these medications were identified in relation to genetically proxied downregulation of IL‐6 signalling [36]. In addition, complement‐related targets (C3 and C5) were identified, with pharmacological inhibitors such as AMY‐101 reported for C3 modulation [25, 26], reflecting the involvement of the innate immune system. Another paper [37] reported GDF15 as a significant target associated with periodontitis and listed several interacting compounds, including sulindac sulfide, resveratrol, indole‐3‐carbinol, and conjugated linoleic acids, which are primarily linked to inflammatory and metabolic regulatory pathways. Other studies identified lipid‐lowering targets such as NPC1L1 and PCSK9, linked respectively to ezetimibe and PCSK9 inhibitors [28]. Furthermore, members of the S100 protein family (S100A8, S100A9, S100A12) and related inflammatory mediators were associated with candidate agents such as colchicine and dexamethasone [31], drugs commonly used for immuno‐inflammatory diseases. More recent integrative studies also proposed verapamil and safinamide as candidate drugs targeting CACNB4 [33], and probenecid, L‐proline, D‐arginine, and sitagliptin in relation to OAT and DPP8 [35]. In contrast, the proteome‐guided study by Alayash et al. identified FGF2, AZGP1, and BTC as potential targets, although no direct approved drug target for periodontitis was identified [27].

**TABLE 2:** Gene/protein‐level drug targets summary.

Gene/Protein	OR (95% CI)	*p*	Gene–environment equivalence for pharmacological translation	Candidate drugs	References
TNFR1 (TNFRSF1A)	1.57 (0.38–6.46)	0.53	Questionable	Etanercept, infliximab, adalimumab, golimumab, certolizumab	[24]
Complement C3	0.91 (0.865–0.958)^a^	< 0.001	Questionable	AMY‐101	[25]
Complement C5	0.89 (0.80–0.98)^a^	0.023	Questionable	—	[26]
FGF2	1.06 (1.032–1.082)^a^	0.0002 (after FDR adjustment)	Not assessable	—	[27]
AZGP1	1.12 (1.058–1.189)^a^	0.0037 (after FDR adjustment)	Not assessable	—	[27]
BTC	0.86 (0.789–0.942)^a^	0.0202 (after FDR adjustment)	Not assessable	—	[27]
HMGCR	0.92 (0.73–1.16)	0.4905	Plausible	—	[28]
NPC1L1	0.53 (0.35–0.81)	0.0038	Plausible	Ezetimibe	[28]
PCSK9	0.84 (0.74–0.95)	0.0051	Plausible	PCSK9 inhibitors	[28]
S100A12	1.31 (1.13–1.52)^a^	< 0.001	Questionable	Miglitol, Labetalol, Phorbol 12‐myristate 13‐acetate	[31]
S100A9	1.09 (1.04–1.14)^a^	< 0.001	Questionable	Chelidonine, Colchicine, Dexamethasone, Mancozeb, Miglitol, Norepinephrine, Phorbol 12‐myristate 13‐acetate, Zinc	[31]
S100A8	0.88 (0.83–0.93)^a^	< 0.001	Questionable	Chelidonine, Colchicine, Dexamethasone, Norepinephrine, Zinc	[31]
ADAM12	1.17 (1.09–1.24)^a^	< 0.001	Questionable	Phorbol 12‐myristate 13‐acetate, Zinc	[31]
ADHFE1	1.07 (1.04–1.11)^a^	< 0.001	Not assessable	—	[31]
BLK	1.14 (1.07–1.21)^a^	< 0.001	Questionable	Colchicine, Zinc, MLN‐8054	[31]
HEBP1	0.93 (0.89–0.96)^a^	< 0.001	Not assessable	—	[31]
SERPINE2	0.79 (0.71–0.89)^a^	< 0.001	Questionable	Zinc	[31]
TEK	0.81 (0.73–0.89)^a^	< 0.001	Questionable	Mancozeb, MLN‐8054	[31]
LY86	0.94 (0.92–0.97)^a^	< 0.001	Not assessable	—	[31]
MMEL1	0.90 (0.87–0.94)^a^	< 0.001	Not assessable	—	[31]
S100B	0.92 (0.89–0.96)^a^	< 0.001	Questionable	Norepinephrine	[31]
SPP1	1.07 (1.03–1.11)^a^	< 0.001	Questionable	Colchicine, Labetalol, Dexamethasone, Phorbol 12‐myristate 13‐acetate, Zinc	[31]
TRIB3	1.13 (1.07–1.19)^a^	< 0.001	Questionable	Chelidonine, Dexamethasone, Zinc	[31]
CACNB4	0.83 (0.71–0.97)^a^	0.019	Questionable	Verapamil, Safinamide	[33]
VWA1	0.94 (0.92–0.97)^a^	< 0.001	Not assessable	—	[37]
GDF15	1.05 (1.02–1.07)^a^	< 0.001	Questionable	Sulindac sulfide, Resveratrol, Indole‐3‐carbinol, 3,3′‐diindolylmethane, Conjugated linoleic acids, AF957.	[37]
IL‐6R	0.81 (0.66–0.99)^a^	0.050	Plausible	Tocilizumab, Satralizumab, Sarilumab, Olokizumab	[36]
CXCL10	0.84 (0.76; 0.91)^a^	0.02 (FDR‐corrected)	Not assessable	—	[34]
MYO3B	0.79 (0.71; 0.88)^a^	0.02 (FDR‐corrected)	Not assessable	—	[34]
SLAMF1	1.19 (1.10; 1.28)^a^	0.02 (FDR‐corrected)	Not assessable	—	[34]
OAT	0.96 (0.95–0.98)^a^	0.0000130	Questionable	Probenecid, L‐proline, D‐Arginine	[35]
DPP8	1.13 (1.07–1.19)^a^	0.0000125	Questionable	Sitagliptin	[35]
CXCR4	1.24 (1.08–1.43)	0.002	Plausible	Alclometasone	[38]
PLAT	1.16 (1.05–1.28)	0.004	Questionable	Alclometasone	[38]
ARHGDIB	1.08 (1.00–1.17)	0.044	Not assessable	—	[38]
C19orf10	1.13 (1.03–1.24)	0.009	Not assessable	—	[38]
VEGFA	1.043 (1.000–1.089)	0.049 (not multiple‐testing corrected)	Questionable	Bevacizumab (Avastin)	[32]
CASP3	1.25 (1.09–1.43)	< 0.001	Questionable	Aspirin	[30]
CCND1	1.55 (1.33–1.81)	< 0.001	Questionable	Aspirin	[30]
MMP25	1.13 (1.07–1.19)	0.019 (FDR‐adjusted)	Not assessable	MMP25 inhibitors (not specified)	[29]

Abbreviations: CASP3, Caspase 3; CCND1, Cyclin D1; GDF15, Growth differentiation factor 15; IL, Interleukin; IVW, Inverse variance weighted; MMP25, Matrix metallopeptidase 25; MR, Mendelian randomisation; TNF, Tumour necrosis factor; VEGFA, Vascular endothelial growth factor A; VWA1, Von Willebrand factor A domain‐containing 1.

^a^ ORs were derived using inverse variance weighting (IVW) unless otherwise specified. ORs from ref. [24] and ref. [38] were obtained using the Wald ratio method. ORs from ref. [28] were obtained using generalised weighted least squares pooled estimates. Gene–environment equivalence plausibility was judged qualitatively as plausible, questionable, or not assessable and was assessed at the target level and refers to whether the direction, mechanism, tissue context, magnitude, and time course of the genetic perturbation could reasonably approximate those of the proposed pharmacological intervention. “Not assessable” indicates insufficient information rather than evidence of implausibility.

The six studies from the Greifswald and Münster groups presented focused analyses on specific potential drug targets. Nolde et al. [36] selected 52 genetic variants in close vicinity to the gene encoding IL‐6 receptor, which were associated with lower circulating C‐reactive protein (CRP). They showed that genetically proxied downregulation of both IL‐6 signalling and CRP was associated with lower odds of periodontitis, suggesting that downregulation of IL‐6 signalling, potentially via IL6RA antagonist, could potentially be a viable drug target. The second paper [26] suggested that downregulation of C3 lowered the odds of having periodontitis in the population, proposing a beneficial effect of pharmacologically targeting C3 for prevention of periodontitis. The third paper [25] showed that inhibiting C5 reduced the risk of periodontitis, while the secondary analysis suggested an involvement of IL‐17 within the potential causal pathway, but was inconclusive for other investigated biomarkers. In contrast, Alayash et al. [24] found no evidence that genetically proxied TNFR1 inhibition reduced periodontitis risk. More recently, Alayash et al. [27] carried out a proteome‐guided drug target discovery analysis and identified three circulating proteins, FGF2, AZGP1, and BTC, as showing robust evidence of causal association with periodontitis. In the same research setting, Baumeister et al. [28] assessed lipid‐lowering drug targets and showed that genetically proxied inhibition of NPC1L1 and PCSK9 was associated with lower odds of periodontitis, whereas HMGCR inhibition was not.

The Hong Kong group [37] analysed 1722 circulating proteins to identify prospective drug targets for periodontitis and conducted a Two‐sample MR to evaluate the bidirectional relationship between circulating proteins and periodontitis risk using data from the FinnGen study, validated by a dataset from the UKBiobank. MR analyses found that genetically predicted circulating levels of von Willebrand factor A domain‐containing 1 (VWA1) were inversely associated with periodontitis, while the level of growth differentiation factor 15 might be associated with an increased risk of periodontitis. Therefore, they suggested that plasma proteins like VWA1 and GDF15 may be potentially therapeutic targets for periodontitis.

More extensive analysis of potential drug targets was carried out in several Chinese studies. Houshen et al. [31] assessed expression quantitative trait loci for 2654 druggable genes. Following the colocalization and summary‐data‐based MR (SMR) analysis, they identified a total of 14 potential therapeutic targets. Among them, 3 targets of the same protein family (S100A12, S100A9, and S100A8) were judged as having higher evidence, while 6 therapeutic targets (ADAM12, ADHFE1, BLK, HEBP1, SERPINE2, and TEK) had moderate evidence, and 5 (LY86, MMEL1, S100B, SPP1, and TRIB3) were defined as having convincing evidence. They also assessed potential for on‐target side effects and examined binding between drugs and their respective protein targets.

In a separate pharmacovigilance‐based investigation, Huoshen et al. [32] integrated FAERS signal detection with DrugBank target mapping, cis‐pQTL analyses from the UKB‐PPP and deCODE cohorts, and single‐cell transcriptomics. Five drugs (risedronate, zoledronic acid, pamidronate, amlodipine, and bevacizumab) showed positive pharmacovigilance signals for periodontitis. VEGFA was the only target reaching nominal significance in the cross‐cohort SMR meta‐analysis (OR 1.043, 95% CI 1.000–1.089; *p* = 0.049; not corrected for multiple testing), while subsequent two‐sample MR analyses supported a positive association between genetically proxied VEGFA levels and periodontitis in FinnGen and GLIDE; single‐cell analyses showed prominent VEGFA expression in monocytes and several gingival stromal and immune‐cell populations.

Liu et al. [34] explored druggable genes using expression quantitative trait loci (eQTL) data from 31 684 individuals and performed MR analyses using periodontitis GWAS data from the FinnGen and GLIDE consortia. According to the results, three replicated druggable genes, CXCL10, MYO3B, and SLAMF1, showed causal associations with periodontitis, with CXCL10 and MYO3B demonstrating protective effects and SLAMF1 showing a deleterious association.

Hu et al. evaluated DrugBank‐defined targets of glucocorticoids and non‐steroidal anti‐inflammatory drugs using blood‐derived cis‐eQTLs located within +/−100 kb of the corresponding genes. Higher genetically proxied expression of CASP3 and CCND1 was associated with acute periodontitis (CASP3: OR 1.25, 95% CI 1.09–1.43; CCND1: OR 1.55, 95% CI 1.33–1.81; both *p* < 0.001), and mediation analyses suggested a potential gut‐microbiota‐mediated pathway for CCND1 [30].

Jiang et al. [33] integrated cis‐eQTL‐based drug‐target MR, colocalization, SMR/HEIDI validation, tissue validation, molecular docking, and pharmacological analyses. They identified six genes associated with periodontitis, of which CACNB4 and PSMA4 were supported by colocalization, and CACNB4 emerged as the main prioritised target after experimental validation. Candidate drugs identified for CACNB4 included verapamil and safinamide, both of which attenuated osteoclast differentiation and alveolar bone loss in experimental models.

Lou et al. [35] integrated genome‐wide association data with single‐cell transcriptomic analysis and identified OAT and DPP8 as potential therapeutic targets for periodontitis. Single‐cell analysis showed predominant OAT expression in fibroblasts, particularly in inflammatory and matrix‐remodelling subsets, and candidate drug prediction linked OAT to probenecid, L‐proline, and D‐arginine, while DPP8 was linked to sitagliptin.

Finally, Zhou et al. [38] applied a multi‐omics framework combining bulk and single‐cell RNA sequencing, eQTL, colocalization, and MR analyses. Four hub genes (CXCR4, ARHGDIB, PLAT, and C19orf10) were identified as potential modulators of periodontitis risk. These genes are implicated in immune regulation, coagulation, and cell migration pathways, highlighting possible new therapeutic targets.

The medRxiv preprint by Cheng et al. [29] screened 2288 of 4479 predefined druggable genes using blood‐derived cis‐eQTLs within +/−100 kb and FinnGen R11 chronic periodontitis data. Thirteen genes passed false‐discovery‐rate correction; MMP25 showed the most consistent support across two‐sample MR, SMR/HEIDI, colocalization, and TWAS, whereas TNFRSF18, CDC25B, STK10, and ACVR2B were supported by MR, SMR/HEIDI, and TWAS but not by colocalization.

Several high‐throughput studies applied multiple‐testing correction, most commonly false discovery rate adjustment, when prioritising candidate targets [27, 31, 33, 34, 37], and some further refined these signals through colocalization, replication, SMR/HEIDI analyses, or experimental follow‐up [27, 31, 33, 34, 35, 37, 38]. However, the extent of triangulation varied substantially across studies.

The credibility assessment was based on the Bashir‐informed framework (Table 3), with study‐specific instrument‐selection characteristics reported in Table S3. At the study level, all included analyses were considered conceptually suitable for MR because they used cis‐acting or target‐proximal instruments to proxy druggable gene or protein traits in relation to periodontal outcomes. This broad applicability judgement did not imply that the corresponding genetic perturbations were equivalent to pharmacological interventions: target‐level gene–environment equivalence was evaluated separately in Table 2 and was frequently rated as questionable or not assessable. Instrument‐selection strategies varied substantially across studies. Cis instruments were defined using study‐specific genomic windows around the target gene, together with heterogeneous association thresholds and LD‐handling criteria. Instrument relevance (Q1) was therefore judged individually according to the biological specification of the cis‐region, the selection threshold and its justification, the number and reported strength of the retained instruments, rather than against a uniform genome‐wide significance threshold. LD clumping or modelling of correlated variants was additionally considered under analytical robustness (Q4). Instrument relevance (Q1) was rated Low in seven studies and Some concerns in eight. Low ratings were assigned where the cis‐region, association threshold, LD handling, instrument number, and instrument strength were sufficiently specified, including the focused TNFR1, C3, C5, IL6R, and lipid‐lowering analyses, the proteome‐guided analysis by Alayash et al. [24, 25, 26, 27], and the druggable‐genome analysis by Cheng et al. [29]. By contrast, Q1 raised some concerns in Hu et al. [30] and the pharmacovigilance‐based study by Huoshen et al. [32] because key target‐gene or cis‐pQTL selection parameters were incompletely reported; similar concerns applied to other high‐throughput studies using relaxed thresholds or incompletely described LD procedures. No uniform genome‐wide significance threshold was imposed.

**TABLE 3:** Risk of bias assessment of the included Mendelian randomisation studies.

Study	MR applicability (study level)	Q1 instrument relevance	Q2 exchangeability	Q3 exclusion restriction	Q4 analytical robustness	Q5 reporting/outcome interpretation	Overall judgement	Brief justification
Alayash et al. 2023 [24]	Plausible	Low	Some concerns	Some concerns	Some concerns	Some concerns	High	Focused TNFR1 cis‐MR using a +/−1 kb region, *p* < 5 × 10^−5^ and primary LD clumping at r2 < 0.001; F‐statistics were 54–118. The secondary analysis appropriately modelled three correlated variants (r2 about 0.7), but no colocalisation or independent outcome replication was performed and the GLIDE outcome was heterogeneous.
Alayash et al., 2024 (a) [25]	Plausible	Low	Low	Some concerns	Low	Some concerns	Some concerns	Focused C3 cis‐MR using a +/−300 kb region, *p* < 1 × 10^−4^, r2 < 0.001, three instruments and F‐statistics of 13–45. PhenoScanner and leave‐one‐out analyses supported the design, although colocalisation and independent outcome replication were not performed and the GLIDE outcome combined heterogeneous definitions.
Alayash et al., 2024 (b) [26]	Plausible	Low	Low	Some concerns	Low	Some concerns	Some concerns	Focused C5 cis‐MR using variants within 1 Mb, *p* < 1 × 10^−5^, r2 < 0.1, 26 instruments and F‐statistics > = 10. A relaxed *p* < 1 × 10^−3^ threshold was biologically justified for secondary single‐locus biomarker analyses; leave‐one‐out analyses were reported, but colocalisation and independent outcome replication were absent.
Alayash et al. 2026 [27]	Plausible	Low	Some concerns	Low	Low	Some concerns	Some concerns	Proteome‐wide cis‐pQTL MR using a +/−1 Mb window, a gene‐level *p* < 5 × 10^−6^ threshold, r2 < 0.1, MAF > 1% and F‐statistics > = 10. The principal targets used 102, 28 and 11 variants, respectively, and were supported by replication, FDR control, Steiger filtering, HEIDI and colocalisation; outcome definitions nevertheless differed between MVP and GLIDE
Baumeister et al., 2023 [28]	Plausible	Low	Low	Some concerns	Low	Some concerns	Some concerns	Focused cis‐MR of HMGCR, NPC1L1 and PCSK9 using +/−100 kb windows, *p* < 5 × 10^−8^ and correlated variants with r2 < 0.2 modelled by generalised weighted least squares. Seventeen instruments had F‐statistics of 19.5–4721 and a secondary exposure dataset and leave‐one‐out analyses were used; no colocalisation was performed
Cheng et al., 2025 [29] (medRxiv preprint)	Plausible	Low	Some concerns	Low	Some concerns	Some concerns	Some concerns	Druggable‐genome cis‐eQTL MR using +/−100 kb windows, *p* < 5 × 10^−8^, r2 < 0.1 and 27 496 instruments across 2288 genes; F‐statistics ranged from 29.7 to 3450.7. FDR correction, SMR/HEIDI, colocalisation, TWAS and PheWAS strengthened target prioritisation, but no independent periodontal outcome replication was undertaken and the report remains a preprint.
Hu et al., 2025 [30]	Plausible	Some concerns	Some concerns	Some concerns	Some concerns	Some concerns	High	DrugBank‐defined target genes were proxied by blood cis‐eQTLs within +/−100 kb. However, the target‐gene association threshold, LD criterion, instrument number and strength were not clearly reported separately from the drug‐level analyses. The study lacked colocalisation and external replication and relied on a small registry‐defined acute‐periodontitis outcome.
Huoshen et al., 2025 [31]	Plausible	Low	Some concerns	Some concerns	Low	Some concerns	Some concerns	A curated druggable‐gene screen used cis‐eQTLs within +/−100 kb, r2 < 0.1 and F‐statistics > 10; the exposure‐association threshold for the primary MR was not explicitly stated, while SMR used +/−1 Mb and *p* < 5 × 10^−8^. FDR control, Steiger filtering, colocalisation, SMR/HEIDI, PheWAS and single‐cell analyses improved robustness, but blood‐only eQTLs and GLIDE phenotyping remained limitations.
Huoshen et al., 2026 [32]	Plausible	Some concerns	Some concerns	Some concerns	Some concerns	Some concerns	High	DrugBank targets were evaluated using cis‐pQTLs from UKB‐PPP and deCODE, but the main report did not state the cis‐window, selection threshold, LD criterion, instrument count or F‐statistics. Cross‐cohort SMR and two‐sample MR provided consistency for VEGFA, but the initial meta‐analytic signal was nominal, uncorrected for multiple testing and not supported by colocalisation.
Jiang et al., 2026 [33]	Plausible	Low	Some concerns	Low	Low	Some concerns	Some concerns	The analysis used a deliberately relaxed *p* < 1 × 10^−5^ threshold with all F‐statistics > 10, but the cis‐window, LD‐clumping criterion and target‐specific instrument counts were not reported in the primary article. Replication in GLIDE, colocalisation, SMR/HEIDI, tissue validation and pharmacological experiments supported robustness; FinnGen and GLIDE used different outcome.
Liu et al., 2024 [34]	Plausible	Low	Some concerns	Some concerns	Some concerns	Some concerns	High	Blood cis‐eQTLs were restricted to 5 kb and FDR < 0.05, with r2 = 0.2 in discovery and r2 = 0.001 in validation. The liberal discovery clumping increased instrument availability, but F‐statistics were not reported; colocalisation and pQTL follow‐up were partial and periodontal outcomes differed across FinnGen, GLIDE and UK Biobank resources.
Lou et al., 2026 [35]	Plausible	Low	Some concerns	Some concerns	Some concerns	Some concerns	High	Blood cis‐eQTLs were selected within +/−100 kb at FDR < 0.05 and clumped at r2 = 0.20 over 10 000 kb, with retained instruments required to have F‐statistics > 10. The permissive threshold and LD criterion increased power but raised robustness concerns; no independent outcome replication was performed and the outcome was limited to FinnGen.
Nolde et al., 2023 [36]	Plausible	Low	Low	Some concerns	Low	Some concerns	Some concerns	Focused IL6R cis‐MR used 52 variants within +/−300 kb, *p* < 5 × 10^−8^ and r2 < 0.2, with F‐statistics of 31–1802. Leave‐one‐out and PhenoScanner analyses supported the design, but colocalisation and independent outcome replication were not performed and GLIDE combined heterogeneous case definitions.
Zhan et al., 2024 [37]	Plausible	Low	Some concerns	Some concerns	Some concerns	Some concerns	High	Proteome‐wide MR selected genome‐wide significant cis‐pQTLs within +/−250 kb and required F‐statistics > 10, but the LD‐clumping or correlation‐handling criterion was not reported. External validation, bidirectional analyses, Steiger filtering and cML‐MA were used, although colocalisation was absent and outcome definitions differed between discovery and validation datasets.
Zhou et al., 2026 [38]	Plausible	Some concerns	Some concerns	Some concerns	Some concerns	Some concerns	High	The SMR/eQTL analysis used the top cis‐QTL within +/−1 Mb at *p* < 5 × 10^−8^, but F‐statistics and an LD‐clumping criterion were not reported. Colocalisation, HEIDI, mediation and single‐cell analyses provided complementary evidence, but there was no independent outcome replication and the framework primarily supports shared genetic signals rather than conventional multi‐instrument causal inference.

*Note:* Each domain was judged as *Low*, *High*, or *Some concerns*. The overall RoB judgement was derived from domain‐level assessments (Low = all domains Low or ≤ 1 Some concerns; Some concerns = 2–3 Some concerns or 1 High; High = ≥ 4 Some concerns or ≥ 2 High). MR plausibility was assessed separately and not included in the overall judgement. MR applicability refers to whether genetically proxied modulation of a druggable gene or protein represents a conceptually appropriate exposure for MR analysis in relation to periodontitis. It does not assess whether the lifelong genetic perturbation is equivalent to a pharmacological intervention, which is evaluated separately at the target level in Table 2. Q1 incorporated the biological definition and width of the cis‐region, the instrument‐selection threshold and its justification, the number and reported strength of the instruments. Q4 incorporated LD clumping or other methods used to account for correlation among cis variants. Full study‐specific details are reported in Table S3.

Abbreviation: RoB, Risk of Bias.

Exchangeability (Q2) was rated Low in four focused analyses (C3, C5, lipid‐lowering targets, and IL6R) and Some concerns in the remaining 11 studies. The latter judgement reflected reliance on blood‐derived expression or circulating‐protein proxies, limited direct assessment of confounding pathways, and, in broader scans, the possibility that target‐associated variants captured additional biological effects. Cheng et al. [29] therefore retained some concerns for Q2 despite strong instruments and extensive downstream analyses, while Hu et al. [30] and Huoshen et al. [32] were also rated Some concerns because their blood‐derived or circulating‐protein proxies and limited confounder assessment did not fully establish exchangeability. Exclusion restriction (Q3) was rated Low in only three studies [27, 29, 33], in which colocalization and/or HEIDI‐type analyses provided stronger support against alternative causal variants. The other 12 studies were rated Some concerns, which did not report colocalization, and focused single‐locus studies in which cis restriction and sensitivity analyses reduced but could not eliminate the possibility of horizontal pleiotropy.

Analytical robustness (Q4) was rated Low in seven studies and Some concerns in eight. Low ratings reflected adequate LD handling together with combinations of replication, colocalization or HEIDI analyses, Steiger filtering, sensitivity analyses, or experimental validation, such as in Alayash et al. [27], Baumeister et al. [28], Jiang et al. [33], and Nolde et al. [36], whereas some concerns remained for broader exploratory or multi‐omics studies without external replication or with only partial downstream validation, including Liu et al. [34], Lou et al. [35], and Zhan et al. [37]. Cheng et al. [29] was rated Some concerns despite extensive SMR/HEIDI, colocalization, TWAS, and PheWAS support because no independent periodontal‐outcome replication was undertaken and the report remained a preprint. Hu et al. [30] was rated Some concerns because instrument‐selection details were incomplete and neither colocalization nor external replication was performed. The pharmacovigilance‐based Huoshen study [32] likewise received Some concerns: cross‐cohort cis‐pQTL and two‐sample MR analyses provided consistency for VEGFA, but the initial meta‐analytic signal was nominal, uncorrected for multiple testing, and unsupported by colocalization.

Reporting and outcome interpretation (Q5) was rated Some concerns in all 15 studies because periodontal outcomes were derived from a limited set of heterogeneous GWAS resources. This concern was particularly marked for Hu et al., which used a small registry‐defined acute‐periodontitis outcome, and remained relevant for Huoshen et al. and Cheng et al., whose analyses relied on registry‐ or consortium‐defined periodontal phenotypes rather than uniformly clinically examined disease [24, 25, 26, 27, 28, 29, 30, 31, 32, 33, 34, 35, 36, 37, 38].

Overall, seven studies were judged as having some concerns [25, 26, 27, 28, 29, 33, 36], whereas eight were classified as high concern [24, 30, 31, 32, 34, 35, 37, 38], reflecting the accumulation of concerns across multiple credibility domains rather than the presence of a single critical flaw. No study met the predefined criteria for an overall Low judgement. Focused cis‐drug‐target analyses generally showed greater methodological coherence than broader exploratory or high‐throughput pipelines, but even the stronger studies retained limitations related to untestable MR assumptions, outcome heterogeneity, and the absence of fully independent replication.

Using UniProt‐derived annotations, functional commonality was explored by mapping each identified target to three functional categories (molecular function, biological process, and ligand) and visualising the overlap in a Venn diagram (Figure 2). Across all included studies, several targets clustered within the shared intersection of the three categories, including *S100A8*, *S100A9*, *S100A12*, *SPP1*, *MYO3B*, and *TEK*, suggesting convergence on proteins with multiple annotated roles. Additional genes were distributed across pairwise overlaps (e.g., *CXCL10*, *SERPINE2*, *SLAMF1*, *TRIB3*) or single‐category assignments. As a qualitative sensitivity analysis, repeating the same mapping after restricting to studies judged at overall low risk of bias yielded a similar overlap structure, with the core multi‐category targets remaining represented (Figure 2A). Overall, functional commonality patterns appeared stable after excluding studies with some concerns of bias, supporting the robustness of the main biological themes emerging from Table 4.

**FIGURE 2:**
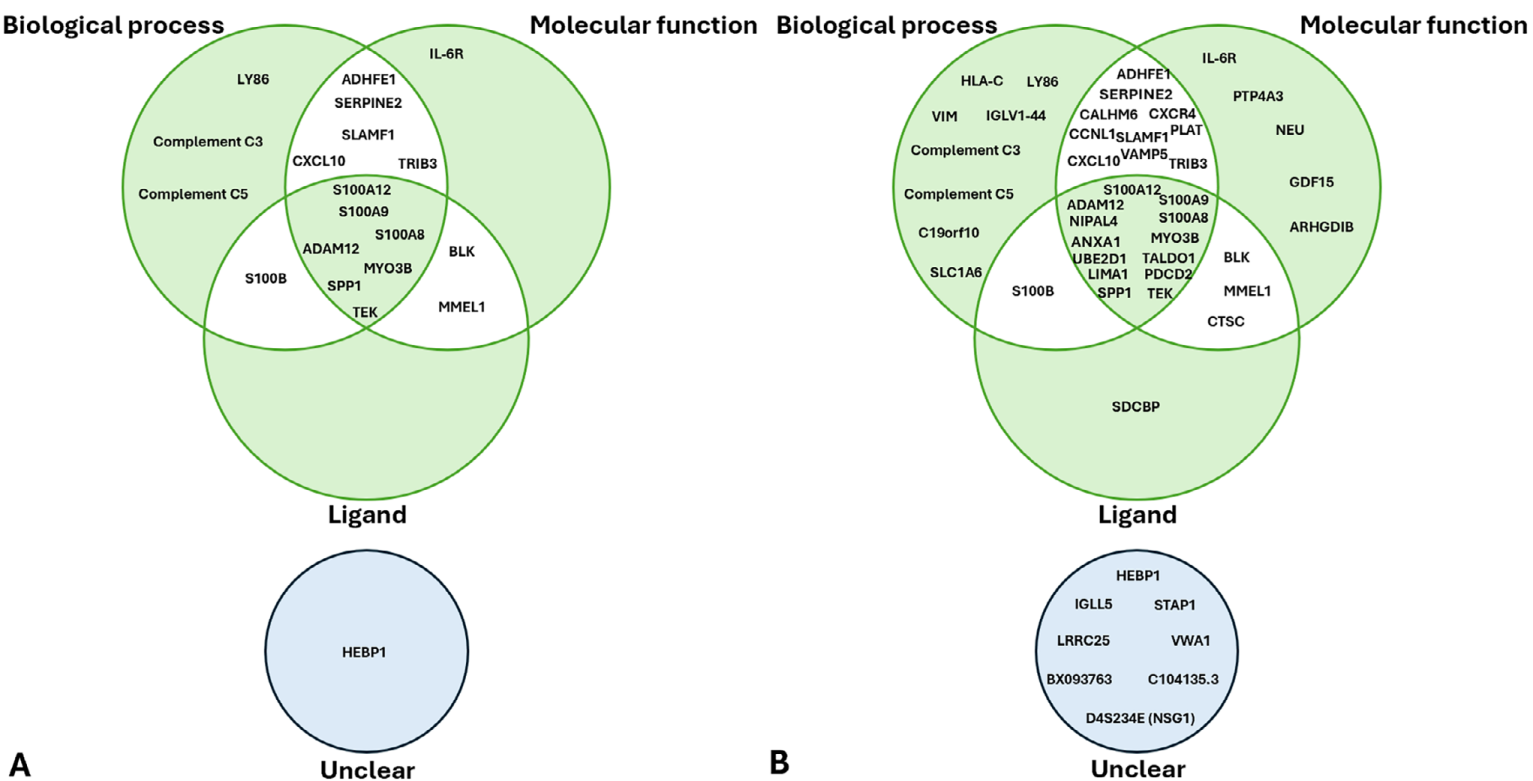
(A) Venn diagram illustrating the overlap between molecular function, biological process, and ligand categories restricted to studies judged at overall low risk of bias. (B) Venn diagram illustrating the overlap between UniProt‐based annotations for molecular function, biological process, and ligand categories across all 12 included MR studies. Genes without annotated functional classification were excluded from the diagram and considered separately as “unclear.”

**TABLE 4:** Biological function and keyword annotation of drug‐target MR genes/proteins.

Gene/Protein	Biological function	Keywords	References
Studies with come concerns			
S100A12	Molecular function Biological process Ligand	#Antibiotic #Antimicrobial #Fungicide #Immunity #Inflammatory response #Innate immunity #Calcium #Copper #Metal‐binding #Zinc	[31]
S100A9	Molecular function Biological process Ligand	#Antimicrobial #Antioxidant #Apoptosis #Autophagy #Chemotaxis #Immunity #Inflammatory response #Innate immunity #Calcium #Metal‐binding #Zinc	[31]
S100A8	Molecular function Biological process Ligand	#Antimicrobial #Apoptosis #Autophagy #Chemotaxis #Immunity #Inflammatory response #Innate immunity #Calcium #Metal‐binding #Zinc	[31]
ADAM12	Molecular function Biological process Ligand	#Hydrolase #Metalloprotease #Protease #Cell adhesion #Metal‐binding #Zinc	[31]
ADHFE1	Molecular function Biological process	#Oxidoreductase #Lipid metabolism	[31]
BLK	Molecular function Ligand	#Kinase #Transferase #Tyrosine‐protein kinase #ATP‐binding #Nucleotide‐binding	[31]
HEBP1	—	—	[31]
SERPINE2	Molecular function Biological process	#Developmental protein #Heparin‐binding #Protease inhibitor #Serine protease inhibitor #Differentiation #Neurogenesis	[31]
TEK	Molecular function Biological process Ligand	#Kinase #Receptor #Transferase #Tyrosine‐protein kinase #Angiogenesis #ATP‐binding #Nucleotide‐binding	[31]
LY86	Biological Process	#Immunity #Inflammatory response #Innate immunity	[31]
MMEL1	Molecular function Ligand	#Hydrolase #Metalloprotease #Protease #Metal‐binding #Zinc	[31]
S100B	Biological process Ligand	#Cell adhesion #Calcium #Metal‐binding #Zinc	[31]
SPP1	Molecular function Biological process Ligand	#Cytokine #Biomineralisation #Cell adhesion #Sialic acid	[31]
TRIB3	Molecular function Biological process	#Protein kinase inhibitor #Apoptosis #Stress response #Transcription #Transcription regulation	[31]
Complement C5	Biological Process	#Complement alternate pathway #Complement pathway #Cytolysis #Immunity #Inflammatory response #Innate immunity	[26]
Complement C3	Biological Process	#Complement alternate pathway #Complement pathway #Fatty acid metabolism #Immunity #Inflammatory response #Innate immunity #Lipid metabolism	[25]
IL‐6R	Molecular function	#Receptor	[36]
FGF2	Molecular function Biological process	#Developmental protein #Growth factor #Heparin‐binding #Mitogen #Angiogenesis #Differentiation	[27]
AZGP1	—	—	[27]
BTC	Molecular function	#Growth factor #Mitogen	[27]
HMGCR	Molecular function Biological process	#Oxidoreductase #Cholesterol biosynthesis #Cholesterol metabolism #Lipid biosynthesis #Lipid metabolism #Steroid biosynthesis #Steroid metabolism #Sterol biosynthesis #Sterol metabolism	[28]
NPC1L1	Biological process	#Cholesterol metabolism #Lipid metabolism #Steroid metabolism #Sterol metabolism #Transport	[28]
PCSK9	Molecular function Biological process Ligand	#Hydrolase #Protease #Serine protease #Apoptosis #Cholesterol metabolism #Lipid metabolism #Steroid metabolism #Sterol metabolism #Calcium	[28]
CACNB4	Molecular function Biological process Ligand	#Calcium channel #Ion channel #Voltage‐gated channel #Calcium transport #Ion transport #Transport #Calcium	[33]
Studies with high risk of bias			
MYO3B	Molecular function Biological process Ligand	#Actin‐binding #Kinase #Motor protein #Myosin #Serine/threonine‐protein kinase #Transferase #Hearing #Sensory transduction #Vision #ATP‐binding #Nucleotide‐binding	[34]
SLAMF1	Molecular function Biological process	#Host cell receptor for virus entry #Receptor #Adaptive immunity #Cell adhesion #Host‐virus interaction #Immunity #Innate immunity #Phagocytosis	[34]
MYO3B	Molecular function Biological process Ligand	#Actin‐binding #Kinase #Motor protein #Myosin #Serine/threonine‐protein kinase #Transferase #Hearing #Sensory transduction #Vision #ATP‐binding #Nucleotide‐binding	[34]
VWA1	—	—	[37]
GDF15	Molecular function	#Cytokine #Hormone	[37]
CXCR4	Molecular function Biological process	#G‐protein coupled receptor #Host cell receptor for virus entry #Receptor #Transducer #Host‐virus interaction	[38]
PLAT	Molecular function Biological process	#Hydrolase #Protease #Serine protease #Plasminogen activation	[38]
ARHGDIB	Molecular function	#GTPase activation	[38]
C19orf10	Biological process	#Angiogenesis #Apoptosis	[38]
TNFR1 (TNFRSF1A)	Biological process	#Apoptosis	[24]
OAT	Molecular function Ligand	#Aminotransferase #Transferase #Pyridoxal phosphate	[35]
DPP8	Molecular function Biological process	#Aminopeptidase #Hydrolase #Protease #Serine protease #Apoptosis	[35]
VEGFA	Molecular function Biological process	#Developmental protein #Growth factor #Heparin‐binding #Mitogen #Angiogenesis #Differentiation	[32]
CASP3	Molecular function Biological process	#Hydrolase #Protease #Thiol protease #Apoptosis	[30]
CCND1	Molecular function Biological process	#Cyclin #Repressor #Cell cycle #Cell division #DNA damage #Transcription #Transcription regulation	[30]
MMP25	Molecular function Ligand	#Hydrolase #Metalloprotease #Protease #Calcium #Metal‐binding #Zinc	[29]

*Note:* Molecular function: Genes in this category encode proteins that perform specific biochemical or molecular activities at the cellular level. Biological process: Genes in this category encode proteins that participate in defined physiological or cellular processes. Ligand: Genes in this category encode proteins that interact with, bind to, or whose activity is modulated by particular molecules.

The overall evidence was considered very low according to GRADE, due to some risk of bias, inconsistency, indirectness and imprecision.

## 4 Discussion

This is the first systematic review, to our knowledge, focusing on drug‐target MR in periodontitis, a relatively novel method used to identify potential therapeutic effects of drugs, which can be used to inform drug development before clinical trials. The included papers differ among them for analytical approaches as well as choice of targets and populations of interest, ranging from a specific single target to a wider analysis of potential gene‐protein targets. The targets range from C3 [25], C5 [26], IL6RA [36], VWA1 and GDF15 [37], S100A12, S100A9 and S100A8, and another longer list of targets with lower level of evidence [31], as well as additional immune‐ and inflammation‐related genes such as CXCL10, CXCR4, SLAMF1, and PLAT [34, 38]. Potential available drugs suggested range from IL6RA antagonists (Tocilizumab, Satralizumab, Sarilumab, Olokizumab), AMY‐101, Chelidonine, Colchicine, Dexamethasone, Alclometasone, Zinc, and several others.

None of the suggested potential drugs is currently recognised as a potential adjunctive agent in the treatment of periodontitis. The findings by Alayash et al. [25] are in agreement with proof‐of‐concept studies of C3 inhibition in pre‐clinical models of periodontitis [40, 41, 42] and with a randomised controlled clinical trial (Phase IIa) of C3 inhibition in patients with gingivitis [43]. Some of the reported IL6RA antagonists and drugs targeting GDF15 have long been suggested as potential adjuncts for the treatment of periodontitis for their anti‐inflammatory and, in some cases, antimicrobial activities. This is the case, for example, for polyphenol Resveratrol [44, 45] and for Tocilizumab [46]. Satralizumab is a monoclonal antibody approved for the treatment of neuromyelitis optica spectrum disorder, which, interestingly, had been indicated in the top 10 predicted drugs for periodontitis based on transcriptomics data [47]. In the latter study, authors used meta‐analysis of two independent RNA‐sequencing data to detect central hubs in protein–protein interactions in periodontitis and to predict the top 10 drugs that may be repurposed for the treatment of periodontitis based on gene expression and pathway data. Pro‐inflammatory cytokines such as IL‐6, IL‐1 Beta, and TLR4 emerged as relevant targets. Alongside Satralizumab, the other top 10 predicted drugs were Denosumab, Interferon Beta 1B, Brodalumab, Resatorvid, Clazakizumab, Gevokizumab, Galanisertib, Mavrilimumab, and Gimsilumab. All these drugs affect inflammatory response or bone resorption; 7 of these 10 are monoclonal antibodies that inhibit the signalling pathways of inflammatory cytokines, and some are already FDA‐approved for the treatment of other NCDs [47]. A recent study integrated atlas of human adult oral and craniofacial tissues, derived from single‐cell RNA sequencing of ~250 000 cells from 70 samples across 13 oral niches [48]. Analysis of tertiary lymphoid structures (TLS) and fibroblasts identified drug candidates targeting these spatial interactions. Peri‐epithelial fibroblasts adjacent to periodontitis sites shared substantial transcriptional overlap with pro‐inflammatory clusters. Immune‐modulating drugs targeting inflammatory fibroblast‐immune interactions such as Sargramostim (acting on GM‐CSF/CSF2) and Satralizumab (IL‐6RA) and immune checkpoint inhibitors such as Basiliximab (IL‐2RA/CD25) and Muromonab (CD3 receptors on T cells) emerged as potential candidates [48]. Among other drugs suggested in studies included in the present systematic review, Colchicine is prevalently used for gout and Behcet's disease but has already shown promise to reduce periodontal tissue destruction in an animal model [49, 50]. Chelidonine is a benzophenanthridine alkaloid derived from *Chelidonium majus* and has recently emerged as a potential agent against *Porphyromonas gingivalis* [51]. Verapamil and safinamide were identified as candidate drugs targeting CACNB4 by Jiang and co‐workers [33], potentially reducing osteoclast differentiation and alveolar bone loss in experimental models. The efficacy of other potential drugs, such as for example AMY‐101 and C5 inhibition in periodontitis, is yet to be assessed in preclinical and clinical trials. A similar degree of caution is warranted for other target nominations emerging from the included studies. Although the association between VEGFA and periodontitis was supported by cis‐pQTL analyses across two proteomic cohorts and by cell‐specific expression data, the effect was nominally significant and was not adjusted for multiple testing [32]. Similarly, CASP3 and CCND1 were identified through blood‐derived cis‐eQTL analyses of DrugBank‐defined anti‐inflammatory targets, but the evidence was restricted to a registry‐based acute periodontitis phenotype and therefore cannot be directly extrapolated to clinically examined chronic periodontitis [30]. MMP25 received broader support across MR, SMR/HEIDI, colocalization, and TWAS analyses; nevertheless, the evidence derives from a non‐peer‐reviewed preprint based on blood eQTLs and a registry‐defined periodontal outcome [29]. Collectively, these findings illustrate that concordance across multiple analytical layers may strengthen target prioritisation, but does not substitute for independent replication, validation in periodontally relevant tissues, or direct experimental and clinical evaluation.

The promise and enthusiasm for this drug‐target MR approach need to be balanced with some inherent difficulties and natural limitations of the method. While MR is a useful tool, it requires underlying datasets with specific properties and has strict assumptions. Results for MR analyses are contingent on the properties of the underlying datasets, and biases or lack of statistical power in these datasets will be visible in downstream MR results. Ideally, these studies require precise, unbiased estimates of the association between genotype and exposure, and between genotype and outcome, obtained in the same underlying population. Precise genotype‐outcome estimates are difficult to obtain, given the properties of existing GWAS in periodontitis, which have known natural limitations in phenotypic resolution and statistical power. In particular, even with false discovery rates correction within each study, the cumulative false positive rate across the entire field is not addressed. Although most large‐scale screening studies used study‐level multiple‐testing correction, usually false discovery rate adjustment, statistical significance alone remains insufficient for target prioritisation. Confidence in candidate targets is strengthened when MR findings are triangulated with independent replication, colocalization, functional validation, or other orthogonal sources of evidence; however, such triangulation was inconsistent across the included studies. The value of MR experiments will grow as the quality and availability of periodontitis GWAS improves.

The assumption of a single underlying population is challenging given all studies used a 2‐sample design with demographic and other differences between populations included in projects such as GTEx and GLIDE. In this respect, it has previously been noticed how most MR studies used outcome data from the GLIDE consortium, which used both self‐reported and clinical measures of periodontitis [23]. Here any misclassification in disease status in either the clinical or self‐reported datasets is anticipated to be uncorrelated with genotype, so is treated as random measurement error in the context of applied genetic epidemiology. Such misclassification, if present, will bias causal estimates towards zero in MR experiments which use periodontitis as an outcome, resulting in both a directional bias in the estimate itself and reduced power in any hypothesis test which aims to detect non‐zero causal effects.

Aside from these strict data requirements, MR makes three classical assumptions. Instrument‐selection strategies also differed markedly across studies in the definition of the cis‐region, association threshold, and LD‐handling procedure. More permissive thresholds may increase the number of available instruments and statistical power, but require explicit biological justification and adequate evaluation of instrument strength because they may increase susceptibility to weak‐instrument and winner's‐curse bias [52]. Although instrument relevance was generally supported in these experiments, the independence and exclusion restriction criteria are strong and difficult to test or satisfy perfectly. MR estimates are often interpreted as ‘life course’ effects of an exposure (given that genotypes are assigned at conception) and mapping these to the effects of a short‐term therapeutic intervention is challenging. This distinction explains why an exposure–outcome pair may be conceptually suitable for MR while its pharmacological translation remains uncertain. MR applicability concerns whether genetic variation can plausibly instrument the molecular exposure, whereas gene–environment equivalence concerns whether the resulting lifelong genetic perturbation adequately represents the direction, magnitude, timing, tissue specificity, and reversibility of a therapeutic intervention. Working with these natural limitations, the presented studies have attempted to design strong analytical strategies and, reassuringly, showed some stability across different sensitivity analyses.

The novelty of this review lies in the systematic assessment of drug‐target MR studies in periodontitis using a credibility framework informed by Bashir et al. [23]. The evidence remains preliminary, largely derived from European and East Asian populations, and affected by phenotype heterogeneity, limited replication, and variable analytical strategies. In the present review, seven studies were judged as having some concerns and eight as high concern, while none fulfilled the criteria for low concern. Instrument relevance was generally well supported, particularly in focused cis‐target designs, whereas exchangeability and exclusion restriction more often remained only biologically plausible rather than directly verifiable. A recurrent limitation was the interpretability of the periodontal outcome, since most studies relied on a small number of GWAS resources, mainly GLIDE and FinnGen, with different case definitions and ascertainment strategies. Here there is natural trade‐off since refined periodontal phenotypes are generally only available in smaller collections (with natural limitations of statistical power) while less‐refined measures are often available in larger resources but are generally more difficult to interpret and prone to measurement error. In addition, several broader multi‐omics or SMR‐based analyses generated potentially interesting targets, but their findings should be interpreted more cautiously than those from focused cis‐drug‐target MR because they often support shared genetic signals rather than standard causal inference. Overall, these findings highlight the promise of drug‐target MR in periodontology, but also the need for better periodontal phenotyping, stronger replication, clearer reporting of MR assumptions, and more functional validation before clinical translation can be considered. The lack of replication of drug targets across included studies may relate to a combination of (non‐exclusive) explanations. These include genuine heterogeneity in effect across studies (i.e., some targets are genuinely more relevant for certain populations), apparent heterogeneity given the different properties of underlying datasets and different analytical designs used and the effects of both false positive and false negative findings given the small causal effects of single protein targets in periodontitis and high multiple testing burden. Although this broadens the range of pathways identified, it also highlights the early and heterogeneous nature of the field, reduces the confidence in these findings and precludes quantitative synthesis based on replicated targets.

Publication and selective‐reporting biases nevertheless cannot be excluded, particularly in high‐throughput target‐screening studies in which statistically significant findings may be preferentially emphasised. This potential bias could not be formally quantified because the included studies evaluated heterogeneous targets and analytical frameworks and no quantitative synthesis was feasible.

In summary, drug‐target MR holds great potential, and we anticipate an explosion of drug‐target MR studies in the near future. While these studies could certainly play an important role in identifying potentially useful drugs, these authors urge caution in the interpretation of the data produced by these studies and recommend strong adherence to rigorous methodology and checklists to minimise the risk of bias in these studies. In particular, assessment of study power is crucial and attention needs to be paid to potential regression dilution bias, independence of assessed populations and penetrance and expressivity. Furthermore, some of the future studies will surely focus on the same underlying datasets, thus not constituting independent lines of evidence. Investment in good‐quality GWAS with clear phenotypes related to clinically relevant data and endpoints is crucial. Furthermore, there is a need for cautious interpretation and consideration of other lines of evidence, such as from functional studies and RCTs, in a triangulation approach. Future MR in this field should focus on large GWAS with adequate periodontal phenotypes and ideally assess disease through genomic datasets in general populations or in the progression/treatment response in periodontitis patients, providing validation in diverse populations and functional characterisation, followed by translational trials assessing safety, efficacy, and cost‐effectiveness before any clinical application can be recommended, particularly if they involve off‐label use of medications designed for the treatment of other disease/conditions [53]. It should also be emphasised that most included studies rely on a limited set of periodontitis GWAS datasets, with inherent limitations potentially affecting the validity and power of MR analyses and assuming that the effect of changing the exposure by genetic variation is equivalent to the effect of changing the exposure by environmental means. It was also recently suggested that triangulating findings from MR studies with results from other methodological approaches is vital to provide a clearer understanding of causal relationships [23, 51]. Furthermore, the possible role of these and other drug targets in periodontology would need to be balanced against costs and adverse events, and their specific application should be clarified (prevention, adjunct to treatment, use in non‐responding cases). At present, antimicrobials remain the drugs with the strongest level of evidence for a role in periodontitis, as adjuncts to mechanical biofilm removal. However, monoclonal antibodies and drugs affecting the inflammatory cytokine cascade and complement activity may in the future be developed or repurposed for the treatment of some periodontitis cases.

## 5 Conclusions

Implications for research: This systematic review identified fifteen Mendelian randomisation studies exploring genetically proxied drug targets for periodontitis. The collective evidence supports the hypothesis that modulating components of the inflammatory cytokine, complement and immune‐cell signalling pathways and extracellular‐matrix remodelling factors could beneficially affect periodontal outcomes. Several candidate targets, including IL6R, C3, C5, CXCL10, CXCR4, and MMP25, have been consistently implicated. Further research is needed in this field, following robust methodology.

Implications for clinical practice: Overall, drug‐target MR provides a powerful framework to prioritise therapeutic candidates for periodontitis, but its results should be interpreted cautiously within the broader context of mechanistic and clinical research, and several steps are needed before clinical applicability.

## Funding

The authors have nothing to report.

## Ethics Statement

The authors have nothing to report.

## Consent

The authors have nothing to report.

## Conflicts of Interest

The authors declare no conflicts of interest.

## Supporting information


**Table S1:** Search Strategy for all the consulted databases.

**Table S2:** Articles excluded during full‐text evaluation.

**Table S3:** Study‐specific instrument‐selection characteristics informing the credibility assessment. Q1 refers to instrument relevance and Q4 to analytical robustness. A permissive selection threshold was not automatically considered problematic; it was interpreted together with biological justification, instrument strength, LD handling, replication, colocalisation and sensitivity analyses. NR, not reported in the primary article; BH, Benjamini‐Hochberg; FDR, false discovery rate; HEIDI, heterogeneity in dependent instruments; IVW, inverse‐variance weighted; LD, linkage disequilibrium; MAF, minor allele frequency; PCA, principal component analysis; pQTL, protein quantitative trait locus; eQTL, expression quantitative trait locus; TSS, transcription start site; TWAS, transcriptome‐wide association study.

**Appendix S1:** PRISMA 2020 Checklist.

## Data Availability

Data available upon request.
