# Supplementary material for: Pharmacological Targets for Periodontitis: A Systematic Review of Drug‐Target Mendelian Randomisation Studies
Source: J Periodontal Res. 2026 Sep 2;61(8):898–928. doi: 10.1111/jre.70168 (PMC13703781; doi:10.1111/jre.70168)
Supplement: Supplementary file 2 [file JRE-61-898-s002.docx]

| **Scientific Databases** | **Search strategy** | **Hits** |
| --- | --- | --- |
| MEDLINE - PubMed  searched on April 14, 2026  [www.ncbi.nlm.nih.gov/sites/entrez/](http://www.ncbi.nlm.nih.gov/sites/entrez/) | ("Periodontitis"[Mesh] OR periodont* OR "periodontal disease") AND ("Mendelian Randomization Analysis"[Mesh] OR "mendelian randomization" OR "mendelian randomisation" OR "MR study" OR "instrumental variable" OR "MR analysis" OR "genetic instruments" OR "causal inference" OR "summary-data-based mendelian randomization" OR SMR OR "colocalization" OR "colocalisation" OR "cis-MR") AND ("drug target" OR "therapeutic target" OR druggable OR "druggable gene" OR "druggable genome" OR "druggable protein" OR "protein target" OR "drug discovery" OR "drug repurposing" OR "target prioritization" OR "target prioritisation" OR protein OR proteins OR "circulating protein" OR "circulating proteins" OR proteome OR "proteome-wide" OR proteomic OR pQTL OR pQTLs OR eQTL OR eQTLs OR "gene expression" OR inhibition OR pharmacogenomics) | 193 |
| WEB OF SCIENCE  searched on April 14, 2026  https://www.webofscience.com/wos/woscc/smart-search | (Periodont* OR "periodontal disease") AND ("Mendelian Randomization Analysis" OR "mendelian randomization" OR "mendelian randomisation" OR "MR study" OR "instrumental variable" OR "MR analysis" OR "genetic instruments" OR "causal inference" OR "summary-data-based mendelian randomization" OR SMR OR "colocalization" OR "colocalisation" OR "cis-MR") AND ("drug target" OR "therapeutic target" OR druggable OR "druggable gene" OR "druggable genome" OR "druggable protein" OR "protein target" OR "drug discovery" OR "drug repurposing" OR "target prioritization" OR "target prioritisation" OR protein OR proteins OR "circulating protein" OR "circulating proteins" OR proteome OR "proteome-wide" OR proteomic OR pQTL OR pQTLs OR eQTL OR eQTLs OR "gene expression" OR inhibition OR pharmacogenomics) | 124 |
| SCOPUS  searched on April 14, 2026  [www.scopus.com](http://www.scopus.com) | (Periodont* OR "periodontal disease") AND ("Mendelian Randomization Analysis" OR "mendelian randomization" OR "mendelian randomisation" OR "MR study" OR "instrumental variable" OR "MR analysis" OR "genetic instruments" OR "causal inference" OR "summary-data-based mendelian randomization" OR SMR OR "colocalization" OR "colocalisation" OR "cis-MR") AND ("drug target" OR "therapeutic target" OR druggable OR "druggable gene" OR "druggable genome" OR "druggable protein" OR "protein target" OR "drug discovery" OR "drug repurposing" OR "target prioritization" OR "target prioritisation" OR protein OR proteins OR "circulating protein" OR "circulating proteins" OR proteome OR "proteome-wide" OR proteomic OR pQTL OR pQTLs OR eQTL OR eQTLs OR "gene expression" OR inhibition OR pharmacogenomics) | 91 |
| **TOTAL** | | **408** |

**Supplementary Table 1**. Search Strategy for all the consulted databases.
