# Supplementary material for: Pharmacological Targets for Periodontitis: A Systematic Review of Drug‐Target Mendelian Randomisation Studies
Source: J Periodontal Res. 2026 Sep 2;61(8):898–928. doi: 10.1111/jre.70168 (PMC13703781; doi:10.1111/jre.70168)
Supplement: Supplementary file 3 [file JRE-61-898-s003.docx]

| **Article** | **Reason for exclusion** |
| --- | --- |
| Baumeister, S.E., et al., Effect of interleukin-17 on periodontitis development: An instrumental variable analysis. Journal of Periodontology, 2023. 94(5): p. 616-621 | Exposure not instrumented as a drug target in a cis-MR framework |
| Czesnikiewicz-Guzik, M., et al., Causal association between periodontitis and hypertension: Evidence from Mendelian randomization and a randomized controlled trial of non-surgical periodontal therapy. European Heart Journal, 2019. 40(42): p. 3459-3470 | Exposure not a drug target |
| Gao, J., Causal Association Between Transferrin Saturation and Periodontitis: A Mendelian Randomization Study. Oral Health Prev Dent, 2026. 24: p. 94-105 | Exposure not a drug target |
| Hui, J.F., et al., Causal effect of chloride intracellular channel protein 5 on chronic periodontitis: A Mendelian randomization study. Medicine, 2026. 105(6) | Exposure not instrumented as a drug target in a cis-MR framework |
| Shi, C.J., et al., Integrating GEO Database, Mendelian Randomization, and Molecular Docking to Identify HLA-C as a Potential Therapeutic Target for Periodontitis. Mediators of Inflammation, 2025. 2025(1) | Exposure not instrumented as a drug target in a cis-MR framework |
| Shi, C.J., et al., Myeloid dendritic cells and periodontal disease association: integrated study of single-cell sequencing and Mendelian randomization analysis. Frontiers in Immunology, 2025. 15 | Exposure not instrumented as a drug target in a cis-MR framework |
| Wang, F., et al., Mendelian randomization analysis identified genes potentially pleiotropically associated with periodontitis. Saudi Journal of Biological Sciences, 2021. 28(7): p. 4089-4095 | Exposure not instrumented as a drug target in a cis-MR framework |
| Wu, Y., et al., The active ingredients and targets of Kouqiangjie formula on periodontitis: a multi-approach study. Naunyn-Schmiedeberg's Archives of Pharmacology, 2025. 398: p. 12519-12541 | Exposure not instrumented as a drug target in a cis-MR framework |
| Xiang, K.Z., C.H. Li, and N. Hu, Identification of Key Periodontitis Genes and Their Mechanisms of Action Using Comprehensive Multiple Microarray Analysis and Mendelian Randomization Methods. International Journal of Genomics, 2025. 2025(1) | Exposure not instrumented as a drug target in a cis-MR framework |
| Yin, C., et al., Senescent Fibroblasts Drive FAP/OLN Imbalance Through mTOR Signaling to Exacerbate Inflammation and Bone Resorption in Periodontitis. Advanced Science, 2025. 12(7): p. 2409398 | Exposure not instrumented as a drug target in a cis-MR framework |
| Zou, M.R. and J.C. Yang, Identify novel therapeutic targets for type II diabetes and periodontitis: insights from single-cell analysis and Mendelian randomization analysis. Frontiers in Endocrinology, 2024. 15 | Exposure not instrumented as a drug target in a cis-MR framework |

**Supplementary Table 2**. Articles excluded during full-text evaluation
