# Supplementary material for: Pharmacological Targets for Periodontitis: A Systematic Review of Drug‐Target Mendelian Randomisation Studies
Source: J Periodontal Res. 2026 Sep 2;61(8):898–928. doi: 10.1111/jre.70168 (PMC13703781; doi:10.1111/jre.70168)
Supplement: Supplementary file 4 [file JRE-61-898-s004.docx]

**Supplementary Table 3. Study-specific instrument-selection characteristics informing the credibility assessment.** Q1 refers to instrument relevance and Q4 to analytical robustness. A permissive selection threshold was not automatically considered problematic; it was interpreted together with biological justification, instrument strength, LD handling, replication, colocalisation and sensitivity analyses. NR, not reported in the primary article; BH, Benjamini-Hochberg; FDR, false discovery rate; HEIDI, heterogeneity in dependent instruments; IVW, inverse-variance weighted; LD, linkage disequilibrium; MAF, minor allele frequency; PCA, principal component analysis; pQTL, protein quantitative trait locus; eQTL, expression quantitative trait locus; TSS, transcription start site; TWAS, transcriptome-wide association study.

| **Study** | **Target / instrument type** | **Cis-window** | **Instrument-selection threshold** | **LD clumping / correlation handling** | **Number of instruments** | **Instrument strength** | **Implications for Q1 and Q4** |
| --- | --- | --- | --- | --- | --- | --- | --- |
| Alayash et al., 2023 | TNFR1; CRP-associated cis variants at TNFRSF1A | ±1 kb | p<5×10^-5 (primary) | r²<0.001 (primary); three secondary variants with r²≈0.7 modelled using correlated-IVW/PCA | 1 primary; 3 secondary | F=54–118 | Q1 Low: narrow biologically specified window and strong instruments. Q4 Some concerns: correlated secondary instruments were modelled, but no colocalisation or independent outcome replication. |
| Alayash et al., 2024 (C3) | Serum C3 cis variants | ±300 kb around C3 locus | p<1×10^-4 | r²<0.001 | 3 | F=13–45 | Q1 Low: explicit cis-window, threshold and acceptable strength. Q4 Low: conservative LD handling, PhenoScanner and leave-one-out analyses; no independent replication. |
| Alayash et al., 2024 (C5) | Plasma C5 cis variants | Within 1 Mb of C5 coding region | p<1×10^-5 (primary); p<1×10^-3 for secondary downstream-biomarker analyses | r²<0.1 | 26 primary; 1–2 per downstream biomarker in secondary analyses | All F≥10 | Q1 Low: relaxed secondary threshold was explicitly justified for a single locus and instruments remained strong. Q4 Low: independent variants and leave-one-out analyses; no colocalisation or independent replication. |
| Alayash et al., 2026 | Proteome-wide circulating-protein cis-pQTLs | ±1 Mb from transcription site | p<5×10^-6 (gene-level threshold); BH-FDR for MR results | r²<0.1; MAF>1% | FGF2 102; AZGP1 28; BTC 11 | F≥10; R² also evaluated | Q1 Low: threshold, window, LD and strength explicitly reported. Q4 Low: replication, FDR, Steiger filtering, HEIDI and colocalisation. |
| Baumeister et al., 2023 | HMGCR, NPC1L1 and PCSK9 variants associated with LDL cholesterol | ±100 kb | p<5×10^-8 | r²<0.2; correlation modelled by generalised weighted least squares | HMGCR 6; NPC1L1 5; PCSK9 6 | F=19.5–4721 | Q1 Low: strong instruments and explicit selection. Q4 Low: correlation modelled, secondary exposure dataset and leave-one-out analyses. |
| Cheng et al., 2025 (preprint) | Blood cis-eQTLs for predefined druggable genes | ±100 kb from TSS | p<5×10^-8 | r²<0.1 | 27,496 instruments across 2,288 genes; target-specific counts not reported in main text | F=29.7–3450.7 | Q1 Low: clear window, threshold, LD and strong instruments. Q4 Some concerns: extensive SMR/HEIDI, colocalisation, TWAS and PheWAS, but no independent periodontal-outcome replication and preprint status. |
| Hu et al., 2025 | DrugBank-defined anti-inflammatory target-gene cis-eQTLs | ±100 kb | NR separately for target-gene analysis; drug-level and mediation analyses used p<1×10^-6 | NR separately for target-gene analysis; drug-level and mediation analyses used r²<0.001 and 10,000-kb clumping window | NR | NR | Q1 Some concerns: key target-gene selection and strength details were incompletely reported. Q4 Some concerns: no colocalisation or external replication; small acute-periodontitis outcome. |
| Huoshen et al., 2026 | DrugBank target cis-pQTLs from UKB-PPP and deCODE | NR in primary report | NR in primary report | NR in primary report | NR; 34 targets mapped and 6 were consistent across both pQTL resources | NR | Q1 Some concerns: cis-pQTL source stated, but window, threshold, LD, count and strength were not reported in the main article. Q4 Some concerns: cross-cohort analyses supported VEGFA, but the initial SMR meta-analysis was nominal and uncorrected, without colocalisation. |
| Huoshen et al., 2025 | Blood cis-eQTLs for a curated druggable-gene set | ±100 kb (primary MR); ±1 Mb (SMR) | Primary exposure threshold NR; MR results controlled at FDR<0.05; SMR p<5×10^-8 | r²<0.1; 10,000-kb distance | Variable; eQTLs available for 2,654 genes | F>10 | Q1 Some concerns: primary exposure threshold not explicit. Q4 Low: Steiger filtering, FDR, colocalisation, SMR/HEIDI, PheWAS and single-cell follow-up. |
| Jiang et al., 2026 | Druggable-gene cis-eQTLs | NR in primary report; detailed procedures in Supporting Information | p<1×10^-5 (explicitly relaxed from 5×10^-8); FDR for discovery results | NR in primary report | Variable; target-specific counts reported in Supporting Information | All F>10 | Q1 Some concerns: relaxed threshold was justified and instruments were strong, but the cis-window and LD criterion were not available in the primary report. Q4 Low: replication, colocalisation, SMR/HEIDI, tissue and pharmacological validation. |
| Liu et al., 2024 | Blood cis-eQTLs for druggable genes | Within 5 kb of target gene | FDR<0.05 | r²=0.2 in discovery; r²=0.001 in validation | Variable across 2,580 genes | NR | Q1 Some concerns: permissive exposure threshold and absent strength metrics. Q4 Some concerns: stricter validation clumping and replication were used, but discovery LD was liberal and downstream validation was partial. |
| Lou et al., 2026 | Blood cis-eQTLs for druggable genes | ±100 kb from TSS | FDR<0.05 | r²=0.20; 10,000-kb clumping window | Variable; target-specific counts NR | F>10 | Q1 Some concerns: permissive threshold and LD criterion despite acceptable F-statistics. Q4 Some concerns: multiple sensitivity analyses and colocalisation, but no independent outcome replication. |
| Nolde et al., 2023 | CRP-associated cis variants at IL6R | ±300 kb | p<5×10^-8 | r²<0.2 | 52 | F=31–1802 | Q1 Low: explicit selection and strong instruments. Q4 Low: leave-one-out and PhenoScanner analyses; no colocalisation or independent replication. |
| Zhan et al., 2024 | Proteome-wide circulating-protein cis-pQTLs | ±250 kb from TSS | Genome-wide significance (exact numerical threshold not restated) | NR | Variable across 1,722 proteins | All F>10 | Q1 Some concerns: strong instruments and clear cis-window, but LD handling was not reported. Q4 Some concerns: external validation, bidirectional analyses, Steiger filtering and cML-MA, but no colocalisation and incomplete LD reporting. |
| Zhou et al., 2025 | Top blood cis-QTL/eQTL for druggable genes | ±1 Mb | p<5×10^-8 | No clumping criterion reported; HEIDI used to assess heterogeneity/linkage | 1 top cis-QTL per gene for Wald-ratio analysis | NR | Q1 Some concerns: no strength metric or LD-clumping criterion. Q4 Some concerns: HEIDI and colocalisation were performed, but no independent outcome replication and the design relied mainly on single-variant SMR/Wald-ratio inference. |
